# Supplementary material for: Roof renewal disparities widen the equity gap in residential wildfire protection
Source: Nat Commun. 2025 Jan 7;16:463. doi: 10.1038/s41467-024-55705-w (PMC11707273; doi:10.1038/s41467-024-55705-w)
Supplement: Supplementary file 1 — Supplementary Information [file 41467_2024_55705_MOESM1_ESM.pdf]

SUPPLEMENTARY INFORMATION for:

Roof renewal disparities widen the equity gap in residential wildfire protection

Supplementary Note 1: Wildfire Grants 2
Supplementary Note 2: Expanded Literature Review 4
Supplementary Note 3: Destroyed Structures in California and the US over Time 7
Supplementary Note 4: Regression Tables and Robustness Checks for Fire Treatment on Roofing Renewals 8
Supplementary Note 5: Roofing Benefits 22
Supplementary Note 6: Exposure projection by States 25
Supplementary Note 7: Firewise Communities analysis 26
Supplementary Note 8: Sample Overview 28
Supplementary Note 9: Dataset Construction (Permits Database and Measuring Distance to Fire) 30
Supplementary Note 10: Identification of a treatment effect 32
Supplementary Note 11: Extended Methods for Counterfactual Simulation 34
Supplementary Note 12: Counterfactual Simulation - Buildings saved vs. Costs 38
Supplementary Note 13: Interaction of fire-resistant roof and construction year 39
Supplementary Note 14: Using California CalEnviroScreen Score instead of Justice40 initiative measurement 40
Supplementary Note 15: Influence of large fire events 42
Supplementary Note 16: Adjusting the roof renewal rates for the share of mobile homes... 44
Supplementary Note 17: Assessing the effect of the margin of error of the ACS median income estimates 45
Supplementary References 47

Supplementary Note 1: Wildfire Grants

Name of Organization	Supported Actions	Type	Funding per Year	Source
Cal Fire	Multiple Options:<br>- Business and Workforce Development<br>- Forest Health & Forest Health Research<br>- Urban and Community Forestry<br>- **Wildfire Prevention** (Vegetation Management, Planning, Education)<br>- **Wildfire Resilience** (Land management, Reforestation)	 E<br>V<br>V<br>D,E,V,<br>P<br><br>V	113 million USD	https://www.fire.ca.gov/what-we-do/grants/wildfire-prevention-grants; Accessed on 14.09.2023
California Forest Improvement Program	- Reforestation<br>- Stand Improvement (Pruning, Thinning)<br>- Forestland conservation practices	V<br>V<br>V	12 – 24 million USD	https://www.fire.ca.gov/what-we-do/grants/california-forest-improvement ; Accessed on 14.09.2023
California Fire Safe Council	- Community Wildfire Protection Plans<br>- Vegetation Management<br>- Prevention education	P<br>V<br>E	1.3 million USD	https://s42264.pcdn.co/wp-content/uploads/2023/07/21-SFA-Grant-Report.pdf ; Accessed on 14.09.2023
California Wildfire Mitigation Program	- Retrofit homes that are at high risk of wildfires (incl. new roofs)<br>- Focus on socially vulnerable communities in San Diego	D,HR	24 million USD *Currently in Pilot Phase. Up to 40k per house*	https://news.caloes.ca.gov/california-sets-framework-for-wildfire-home-hardening-program/ ; Accessed on 14.09.2023
California Fire Foundation	- Vegetation management & Fuels Reduction Education<br>- Planning or Community Outreach Campaigns	V,E<br><br>P	2 million USD	https://www.cafirefoundation.org/what-we-do/for-grant-seekers/what-we-fund ; Accessed on 14.09.2023
**Local / County Initiatives (Selection)**	Home Hardening & Vegetation management			
Marin Wildfire Prevention Authority	- Home Hardening, with focus on vents, garage doors<br>- Defensible Space	HNR<br><br>V	up to 5,000 USD per house	https://www.smfd.org/preparation-resources/your-home-and-property/defensible-space-home-hardening-grant-programs-mwpa ; Accessed on 14.09.2023
Moraga-Orinda Fire Protection	- Home Hardening, with focus on vents and gutters<br>- Defensible Space	HNR<br><br>V	up to 5,000 USD per house	https://www.mofd.org/our-district/fuels-mitigation-fire-prevention/home-hardening-grant-program ; Accessed on 14.09.2023
City of Agoura Hills	- Home Hardening	HR	up to 1,000	https://www.agourahi

			USD per house	llscity.org/department/city-manager/emergency-services/home-hardening-page; Accessed on 14.09.2023
Yuba Watershed Protection & Fire Safe Council	- Home Hardening, any type	HR	up to 2,000 USD per house	https://yubafiresafe.org/home-hardening-cost-share-program/ ; Accessed on 14.09.2023
Town of Woodside	- Home Hardening, any type<br>- Defensible Space	HR<br>V	up to 3,000 USD per house	https://www.woodsidetown.org/community/defensible-space-and-home-hardening-matching-fund-program; Accessed on 14.09.2023
Sonoma County	- Defensible Space	V	37 million USD	https://www.sonomacountypermits.org/divisions/firepreventionandhazmat/grantprograms; Accessed on 14.09.2023
City of Sleepy Hollow	- Home Hardening<br>- Defensible Space	D,HR<br>V	up to 5,000 USD per house	https://shfpd.specialdistrict.org/grants-and-assistance-programs ; Accessed on 14.09.2023

Supplementary Table 1: Overview of California wildfire grants (non-exhaustive). Grants are classified by Vegetation management (V), Education (E), Planning (P), Home Hardening incl. Roof Renewals (HR) and Home Hardening excl. Roof Renewal (HNR). California annually invests hundreds of millions of dollars in grants for wildfire prevention. Larger funding initiatives primarily focus on vegetation management rather than supporting new roofing projects, which are crucial for disadvantaged areas (DACs). Smaller, local initiatives that do fund new roofs often offer limited funding—up to $5,000 per house—which is insufficient for roof replacements that on average cost around $20,000 in DACs. The pilot phase of the California Wildfire Mitigation Program offers promise for more substantial home hardening projects.

Supplementary Note 2: Expanded Literature Review

Protection motivation theory. The Protection Motivation Theory (PMT)[1] offers a framework for explaining how and why individuals are willing to improve their protection behavior. The theory states that for a change in behavior, the protection motivation needs to reach a certain threshold which is in turn a function of the perception of severity. exposure of the risk, and the coping appraisal of the action. The assessment of the coping mechanism depends on the perceived efficacy and the costs of the protection behavior. Some authors also note the importance of "self-efficacy" in this context, namely the belief that an individual is able to perform the task[2]. The PMT also highlights a unique form of availability bias[3], where individuals tend to exercise heightened caution following life events that alter their risk perception, irrespective of whether the fundamental risk has remained constant. While the PMT is mainly applied in the context of protective health behavior[4], a growing field of literature is exploring its use for the explanation of natural disaster responses.

With respect to wildfires, researchers have examined the PMT as an explanation and found that following wildfires – and especially near-miss events – the perception of risk declines, while the willingness to invest in protective measures increases[5,6]. Risk perception thereby mediates the effects of knowledge and the feeling of responsibility[7]. Still, other studies find no connection between risk perception and the willingness to participate in wildfire mitigation behavior[8]. While these studies and others in this field have focused on interviews or homeowner surveys, also empirical evidence of insurance uptake following flood events points at this type of experiential learning, however with no distinction between socioeconomic groups[9].

Wildfire risk mitigation participation factors. While risk perception is recognized as a crucial factor influencing homeowners' participation in wildfire mitigation actions, the literature reveals a range of other determinants, though consensus on their relative importance has yet to be achieved[5]. The identified factors range from an individual's knowledge regarding wildfires[10,11], past experience with wildfires[12–14], their building characteristics and hence the associated risks and mitigation costs[10] to the composition of the community - namely a lower inequality within a group leads to higher mitigation efforts[15], and also community attachment[16]. Moreover, the socioeconomic status of individuals plays an important role[17,18]. A growing body of literature emphasizes the notion that households of socially vulnerable communities take fewer preventive actions[17,19] and this could be due to a variety of reasons, such as lack of accessible information and sufficient financial incentives[20]. In general, literature suggests that homeowners underestimate the risk of natural disasters and consequently underinvest in protective measures[21,22]. However, all of the above mentioned studies used a combination of case studies or surveys with a limited number of participants. A review article from 2018 regarding the risks and mitigation actions of wildfires found that only 12% of the studies used methods of data collection and analysis other than surveys, interviews and hazard assessments in case study type environments[23].

Difference in wildfire risk exposure by socioeconomics. Not only is the number of wildfires increasing, also the human exposure to the fires is increasing as more people move to the WUI [24]. The primary exposure number – the population residing *within* a fire perimeter in the last 20 years – is estimated to be around 600,000 for the US alone[25]. Nonetheless, not all communities are equally vulnerable and susceptible to wildfires. While some literature notes that the majority of the regions with fire risk are not socioeconomically vulnerable[26–28], researchers have often stressed that disadvantaged groups are disproportionately affected by wildfires - also because vulnerability is often a "compounding process" as these communities might have lower access to the resources needed for recovery[29]. Vulnerability or socioeconomic disadvantages can take many forms and can be described in terms of income, education, race/ethnicity, and age. In the context of our research, we are following the definition developed by the US government through its Justice40 initiative[30] for the identification of DACs (see Introduction). In the literature there are findings of disproportionately effects of wildfires on elderly populations[31], communities of color[26], lower income[28] and generally disadvantaged groups[32]. Also, there is evidence of the two-directional relationship of social vulnerability and wildfire risk[33]. For example, socioeconomic factors can also increase the number of wildfires due to rural abandonment or arson[34].

Response to wildfire events. In recent literature, researchers are turning towards observational data to investigate the effects of smoke or fire exposure on different socioeconomic groups[35,36]. They find that while salience towards smoke events is equally increased for all groups, especially socially vulnerable tend to search less for protective measures, revealing income disparities. In addition, socially vulnerable communities bear most of the costs related to reduced earnings associated with smoke events. Following wildfires or smoke exposure, consumers shift their preferences in terms of purchases (opting for more sustainable products) and voting (opting for more pro-environment friendly voting), a treatment effect mainly observed within non-socially vulnerable communities and Democratic voters[37,38]. One study investigates the mandated building codes in California and their influence on the survival rates of structures[39]. The results show significant vintage effects, meaning that older homes are 16 percentage points more likely to be destroyed. Another study[40] found that state fire protection investments are implicit subsidies to homeowners as they ensure the habitability of an area, underlining the need for individual actions. Also *public* preventive measures increase following wildfires, however predominantly for communities with higher socioeconomic status, highlighting further inequalities between socially and not-socially vulnerable communities[19].

Wildfires and the benefits of a new roof. Embers are the leading cause of building ignitions during wildfires[41,42]. There is consensus in academic research and emergency management, that among a building's components, the roof is considered the most vulnerable[43]. But while the need for fire-resistant roofing is generally accepted, research quantifies its relative importance differently. The efficacy of a roof in resisting fire is not only determined by its material but also by its architectural features, such as vents and eaves, which are located on rooftops [44]. Some studies see the roof as the most important feature of a house and include it with a weight of up to 33% in their house vulnerability index[45]. However other studies consider eaves and vents as more critical elements, with relative risk (RR) values of up to -0.21, while

the RR values for roofs is estimated at -0.12[46]. The problem scales when examining the potential for house-to-house fire spread. Studies have found that a single home with a non-fire-retardant roof can lead to the ignition of up to ten neighboring homes[47]. Such a cascade effect amplifies the overall community risk and necessitates broader preventive measures. Financially, the problem is far from trivial. In the United States, the cost of only replacing those roofs made from more combustible wood material is estimated to exceed $6 billion[48].

Supplementary Note 3: Destroyed Structures in California and the US over Time

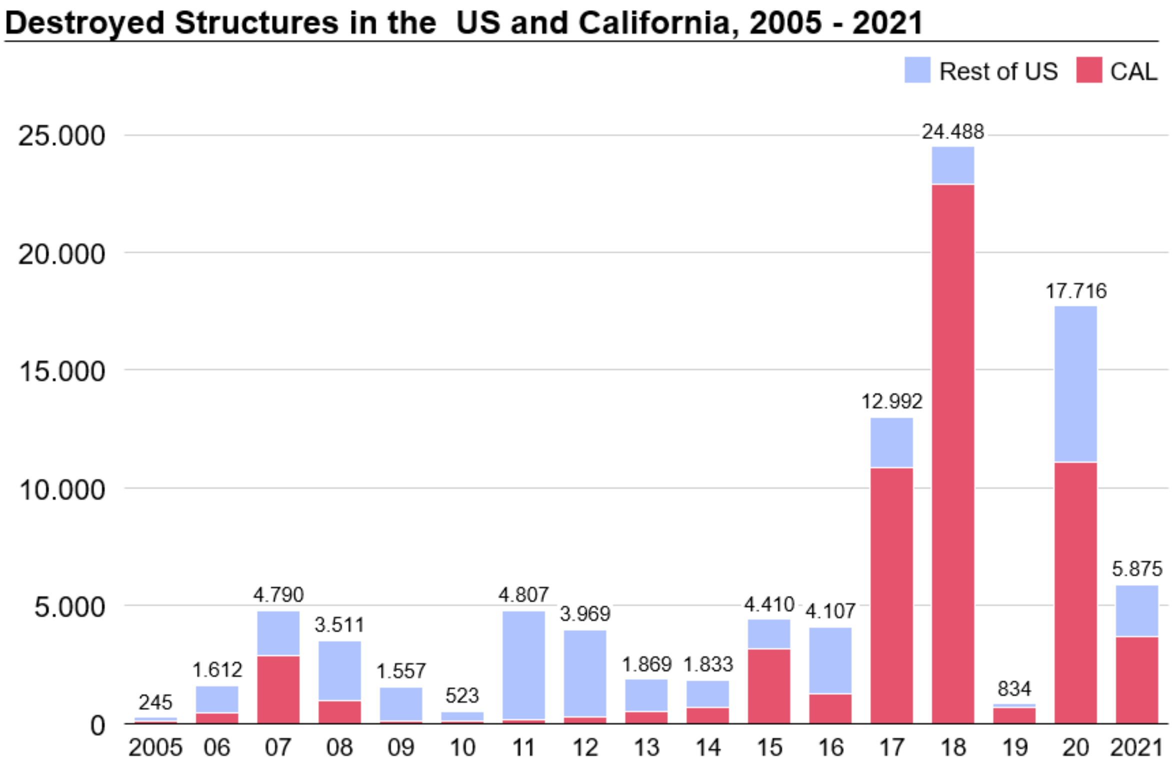

Supplementary Figure 1: Destroyed structures in the US from 2005 to 2021. On average the number of destroyed structures has increased since 2017 partly due highly impactful events, such as the Camp Fire in 2018. In the years 2011 - 2021 an average of 7,536 structures were destroyed, over 65% of which were located in California. Graph is based on statistics provided by CAL FIRE[49] and Headwater Economics[50]. The sample includes *n=95,142* destroyed structures.

Supplementary Note 4: Regression Tables and Robustness Checks for Fire Treatment on Roofing Renewals

We conducted a series of robustness checks to confirm the validity of our findings regarding treatment effects of wildfire exposure on the number of roof renewals. These checks included varying the parameters for proximity and duration of fire impact, redefining the treatment definition, and employing alternative regression models. Across all these variations, our results remain both valid and statistically significant.

Initially, we introduced several control variables and fixed effects (Table 2 and Table 3). Controls included socio-demographic factors, like income, and a variable capturing the previously “Installed Base,” calculated from the American Community Survey (ACS) data on building age as well as the historical building permits, to account for houses that were recently built or had undergone renewal for various reasons, including to meet current building codes. We adjusted for the dynamics of the Wildlife-Urban Interface (WUI) within census tracts, which affect wildfire exposure due to changes in housing growth or vegetation encroachment. We therefore used USGS's WUI estimates and interpolated them on an annual basis[24]. Controls for fire-specific factors were also applied, such as the count of active Firewise communities within a tract and the incidence of minor fires in the preceding three years that fall outside our main treatment definition yet indicate changing fire risks. Additionally, we accounted for fire damage within tracts over the past three years and also explicitly excluded houses that were damaged. This approach ensured that our results reflect preventive renewals rather than mere reconstruction. County-Year fixed effects were also applied to mitigate the impact of regulatory or political shifts across counties.

Next, we varied our treatment definitions (Table 5, Table 6 and Table 7). We replaced our primary binary treatment indicator — the population-weighted distance to wildfires under 10 kilometers within three years — with alternative measurements, including different distances and fire sizes, treatment durations, and other exposure metrics like the number of houses at risk from wildfires. Placebo treatments from two years before a fire were also analyzed, yielding no significant results. We also tested varying cutoffs for ownership levels (Table 8).

Finally, we investigated different model specifications. Therefore we run the regressions using different models such as Poisson, linear regression models, and negative binomial models (Table 9). While the models yield different coefficients, they are similar in direction and magnitude. Still, the fit of the negative binomial regression model for the count of residential roof renewals is better, as confirmed by a likelihood ratio test ($p<0.001$).

Main Treatment Regression

	1	2	3	4	5
Dependent Variable			*No. of Roofing Permits*		
	Coefficient (clustered standard error; t-statistic; P value)	*Coefficient (clustered standard error; t-statistic; P value)*	*Coefficient (clustered standard error; t-statistic; P value)*	*Coefficient (clustered standard error; t-statistic; P value)*	*Coefficient (clustered standard error; t-statistic; P value)*
Wildfire Treated	0.23*** (0.02; 12.28;<0.001)	0.16*** (0.02; 8.51;<0.001)	0.16*** (0.02; 9.18;<0.001)	0.12** (0.04; 2.9;0.004)	0.11*** (0.03; 3.57;<0.001)
Mean Household Income		2.16*** (0.2; 10.9;<0.001)	1.82*** (0.2; 9.17;<0.001)	-0.47 (0.39; -1.21;0.227)	0.17 (0.29; 0.57;0.567)
WUI (Share)			0.35 (0.61; 0.58;0.564)	0.11 (0.5; 0.22;0.825)	-0.11 (0.58; -0.19;0.853)
Moved last 3 years (Share)			-0.97*** (0.08; -12.06;<0.001)	0.5* (0.24; 2.1;0.036)	0.3 (0.18; 1.72;0.086)
Wildfire Damaged Houses			-2.07*** (0.22; -9.41;<0.001)	-2.16*** (0.36; -5.96;<0.001)	-1.58*** (0.32; -4.86;<0.001)
Firewise Communities			0.06** (0.02; 2.86;0.004)	0.04* (0.01; 2.44;0.015)	0.01 (0.01; 0.59;0.557)
Owner Occupied (Share)			-0.08 (0.15; -0.51;0.612)	0.21 (0.21; 0.97;0.332)	0.14 (0.17; 0.87;0.386)
Fixed-Effects:					
Census Tract	*Yes*	*Yes*	*Yes*	*Yes*	*Yes*
Year	*No*	*No*	*No*	*Yes*	*No*
County - Year	*No*	*No*	*No*	*No*	*Yes*
Model Family	Neg. Binom	Neg. Binom	Neg. Binom	Neg. Binom	Neg. Binom
Clustered SE	Tract	Tract	Tract	Tract & Year	Tract & County - Year
Observations	21,688	21,688	21,688	21,688	21,432
R2	0.22	0.23	0.23	0.24	0.26
BIC	129,723	129,43	128,992	128,024	125,014
Over-Dispersion	6.66	6.94	7.39	8.41	13.40

Supplementary Table 2: Negative binomial regression models predicting the number of new roofing permits following a fire treatment. Dependent variable is the number of roofing permits per census tract, winsorized at the 1st and 99th percentile. Standard errors are clustered as denominated and in parentheses. All independent variables are normalized to the [0,1] interval so the coefficients represent the difference in effect size when the corresponding variable is at its maximum (coded as 1) vs. minimum (coded as 0). Significance levels are . p<0.1, * p<0.05, **p<0.01, and ***p<0.001.

DAC Treatment Regression

	1	2	3	4
Dependent Variable	*No. of Roofing Permits*			
	Coefficient (clustered standard error; t-statistic; P value)	*Coefficient (clustered standard error; t-statistic; P value)*	*Coefficient (clustered standard error; t-statistic; P value)*	*Coefficient (clustered standard error; t-statistic; P value)*
DAC Treated	0.13*** (0.04; 3.47;<0.001)	0.04 (0.04; 0.8;0.424)	0.06 (0.04; 1.59;0.112)	0.08* (0.04; 2;0.045)
non-DAC Treated	0.26*** (0.02; 12.44;<0.001)	0.13* (0.06; 2.39;0.017)	0.15** (0.05; 2.71;0.007)	0.11*** (0.03; 3.38;<0.001)
Mean Household Income			-0.49 (0.38; -1.28;0.2)	0.15 (0.29; 0.53;0.597)
WUI (Share)			0.09 (0.5; 0.19;0.85)	-0.11 (0.58; -0.2;0.845)
Moved last 3 years (Share)			0.5* (0.24; 2.09;0.037)	0.3 (0.18; 1.7;0.089)
Wildfire Damaged Houses			-2.14*** (0.37; -5.83;<0.001)	-1.57*** (0.33; -4.83;<0.001)
Firewise Communities			0.03* (0.01; 2.45;0.014)	0.01 (0.01; 0.58;0.56)
Owner Occupied (Share)			0.21 (0.21; 1;0.319)	0.15 (0.17; 0.9;0.37)
Fixed-Effects:				
Census Tract	*Yes*	*Yes*	*Yes*	*Yes*
Year	*No*	*Yes*	*Yes*	*No*
County - Year	*No*	*No*	*No*	*Yes*
Model Family	Neg. Binom	Neg. Binom	Neg. Binom	Neg. Binom
Clustered SE	Tract	Tract & Year	Tract & Year	Tract & County - Year
Observations	21,688	21,688	21,688	21,432
R2	0.22	0.24	0.24	0.26
BIC	129,718	128,166	128,026	125,022
Over-Dispersion	6.68	8.16	8.42	13.41

Supplementary Table 3: Negative binomial regression models predicting the number of new roofing permits following a fire treatment - treatment is split by DAC status. DAC status definition follows the identification by Justice40. Dependent variable is the number of roofing permits per census tract, winsorized at the 1st and 99th percentile. Standard errors are clustered as denominated and in parentheses. All independent variables are normalized to the [0,1] interval so the coefficients represent the difference in effect size when the corresponding variable is at its maximum (coded as 1) vs. minimum (coded as 0). Significance levels are . p<0.1, * p<0.05, **p<0.01, and ***p<0.001.

DAC and Owner Treatment Regression

	1	2	3	4
Dependent Variable	*No. of Roofing Permits*			
	Coefficient (clustered standard error; t-statistic; P value)	*Coefficient (clustered standard error; t-statistic; P value)*	*Coefficient (clustered standard error; t-statistic; P value)*	*Coefficient (clustered standard error; t-statistic; P value)*
DAC * Low Ownership Treated	0.19* (0.08; 2.48;0.013)	0.07 (0.08; 0.78;0.436)	0.07 (0.08; 0.86;0.388)	0.07 (0.08; 0.99;0.324)
Non-DAC * Low Ownership Treated	0.22** (0.08; 2.66;0.008)	0.08 (0.12; 0.69;0.493)	0.09 (0.12; 0.76;0.448)	0.15 (0.11; 1.34;0.179)
DAC * non Low Ownership Treated	0.12** (0.04; 2.81;0.005)	0.03 (0.05; 0.61;0.54)	0.06 (0.04; 1.42;0.157)	0.08 (0.05; 1.72;0.085)
Non-DAC * non Low Ownership Treated	0.26*** (0.02; 12.24;<0.001)	0.14* (0.06; 2.48;0.013)	0.15** (0.05; 2.79;0.005)	0.11*** (0.03; 3.45;<0.001)
Mean Household Income			-0.49 (0.38; -1.3;0.194)	0.15 (0.29; 0.53;0.594)
WUI (Share)			0.09 (0.5; 0.19;0.852)	-0.11 (0.58; -0.2;0.845)
Moved last 3 years (Share)			0.5* (0.24; 2.09;0.037)	0.3 (0.18; 1.71;0.086)
Wildfire Damaged Houses			-2.14*** (0.37; -5.8;<0.001)	-1.57*** (0.33; -4.79;<0.001)
Firewise Communities			0.03* (0.01; 2.45;0.014)	0.01 (0.01; 0.59;0.558)
Owner Occupied (Share)			0.21 (0.22; 0.99;0.322)	0.15 (0.16; 0.9;0.368)
Fixed-Effects:				
Census Tract	*Yes*	*Yes*	*Yes*	*Yes*
Year	*No*	*Yes*	*Yes*	*No*
County - Year	*No*	*No*	*No*	*Yes*
Model Family	Neg. Binom	Neg. Binom	Neg. Binom	Neg. Binom
Clustered SE	Tract	Tract & Year	Tract & Year	Tract & County - Year
Observations	21,688	21,688	21,688	21,432
R2	0.22	0.24	0.24	0.26
BIC	129,737	128,185	128,045	125,041
Over-Dispersion	6.68	8.16	8.42	13.41

Supplementary Table 4: Negative binomial regression models predicting the number of new roofing permits following a fire treatment - treatment is split by DAC status and ownership status. DAC status definition follows the identification by Justice40, while “Low Ownership” is based on ACS estimate of owners vs. renters in a census tract. Low ownership is defined as less than the 25th percentile of owner occupied houses and high ownership as above the 75th percentile. Dependent variable is the number of roofing permits per census tract, winsorized at the 1st and 99th percentile. Standard errors are clustered as denominated and in parentheses. All independent variables are normalized to the [0,1] interval so the coefficients represent the difference in effect size when the corresponding variable is at its maximum (coded as 1) vs. minimum (coded as 0). Significance levels are . p<0.1, * p<0.05, **p<0.01, and ***p<0.001.

221 Robustness Checks - Treatment definition (1)

	1	2	3	4	5	6	7	8	9
Dependent Variable	*No. of Roofing Permits*								
	Coefficient (clustered standard error; t-statistic; P value)	*Coefficient (clustered standard error; t-statistic; P value)*	*Coefficient (clustered standard error; t-statistic; P value)*	*Coefficient (clustered standard error; t-statistic; P value)*	*Coefficient (clustered standard error; t-statistic; P value)*	*Coefficient (clustered standard error; t-statistic; P value)*	*Coefficient (clustered standard error; t-statistic; P value)*	*Coefficient (clustered standard error; t-statistic; P value)*	*Coefficient (clustered standard error; t-statistic; P value)*
**Original Treatment**	0.13** (0.04; 3.17;0.002)								
Number of Houses Treated		0.57** (0.21; 2.68;0.007)							
Treatment Distance 1k			0.15*** (0.04; 3.53;<0.001)						
Treatment Distance 2.5k				0.11** (0.04; 3.12;0.002)					
Treatment Distance 5k					0.14*** (0.04; 4;<0.001)				
Treatment Distance 20k						0.1* (0.04; 2.41;0.016)			
Treatment Distance 50k							-0.11** (0.03; -3.03;0.002)		
Placebo Treatment								0 (0.03; 0.12;0.901)	
Smoke Treatment									0.03 (0.1; 0.3;0.762)
Mean Household Income	-0.92 (0.47; -1.93;0.053)	-0.94* (0.47; -1.99;0.047)	-0.84 (0.49; -1.7;0.09)	-0.85 (0.49; -1.73;0.084)	-0.85 (0.48; -1.76;0.079)	-1.01* (0.48; -2.09;0.036)	-0.65 (0.46; -1.43;0.153)	-0.84 (0.49; -1.71;0.088)	-0.87 (0.49; -1.78;0.076)
WUI (Share)	0.55 (0.75; 0.73;0.465)	0.48 (0.76; 0.63;0.526)	0.62 (0.81; 0.77;0.44)	0.6 (0.8; 0.76;0.449)	0.57 (0.78; 0.73;0.468)	0.61 (0.8; 0.77;0.442)	0.56 (0.79; 0.72;0.472)	0.61 (0.8; 0.76;0.447)	0.61 (0.79; 0.77;0.44)
Installed Base	0.75** (0.27; 2.77;0.006)	0.77** (0.28; 2.8;0.005)	0.81** (0.28; 2.86;0.004)	0.81** (0.28; 2.84;0.005)	0.79** (0.28; 2.84;0.005)	0.73** (0.26; 2.74;0.006)	0.79** (0.27; 2.88;0.004)	0.81** (0.28; 2.94;0.003)	0.83** (0.29; 2.81;0.005)
Moved last 3 years (Share)	0.39 (0.29; 1.36;0.174)	0.38 (0.29; 1.3;0.193)	0.38 (0.31; 1.24;0.216)	0.38 (0.3; 1.24;0.215)	0.37 (0.3; 1.25;0.21)	0.39 (0.3; 1.3;0.194)	0.37 (0.29; 1.27;0.205)	0.38 (0.31; 1.23;0.219)	0.39 (0.31; 1.25;0.21)
Wildfire Damaged Houses	1.86*** (0.54; 3.47;<0.001)	1.95*** (0.53; 3.69;<0.001)	1.85*** (0.52; 3.55;<0.001)	1.86*** (0.52; 3.57;<0.001)	1.82*** (0.53; 3.45;<0.001)	1.95*** (0.52; 3.72;<0.001)	1.91*** (0.5; 3.8;<0.001)	1.94*** (0.51; 3.83;<0.001)	1.95*** (0.51; 3.82;<0.001)
Firewise Communities	0.03* (0.01; 2.18;0.03)	0.03 (0.01; 1.74;0.082)	0.03* (0.01; 2.16;0.031)	0.03* (0.01; 2.17;0.03)	0.03* (0.01; 2.18;0.029)	0.03* (0.01; 2.18;0.03)	0.03 (0.01; 1.81;0.07)	0.03* (0.01; 2.06;0.04)	0.03 (0.02; 1.81;0.071)
Owner Occupied (Share)	0.33 (0.21; 1.6;0.11)	0.33 (0.21; 1.6;0.11)	0.28 (0.21; 1.32;0.185)	0.29 (0.21; 1.36;0.173)	0.3 (0.21; 1.43;0.154)	0.33 (0.21; 1.59;0.111)	0.27 (0.22; 1.24;0.215)	0.28 (0.21; 1.32;0.186)	0.28 (0.22; 1.31;0.191)
Count of Small Fires (Log)	0.31 (0.16; 1.92;0.055)	0.3 (0.16; 1.86;0.062)	0.33* (0.16; 2;0.045)	0.33* (0.16; 2;0.045)	0.32* (0.16; 1.98;0.048)	0.28 (0.16; 1.68;0.093)	0.37* (0.18; 2.14;0.032)	0.33* (0.17; 2.01;0.045)	0.33* (0.16; 2.01;0.045)
Fixed-Effects:									
Census Tract	*Yes*	*Yes*	*Yes*	*Yes*	*Yes*	*Yes*	*Yes*	*Yes*	*Yes*
Year	*Yes*	*Yes*	*Yes*	*Yes*	*Yes*	*Yes*	*Yes*	*Yes*	*Yes*
Model Family	Neg. Binom	Neg. Binom	Neg. Binom	Neg. Binom	Neg. Binom	Neg. Binom	Neg. Binom	Neg. Binom	Neg. Binom
Clustered SE	Tract & Year	Tract & Year	Tract & Year	Tract & Year	Tract & Year	Tract & Year	Tract & Year	Tract & Year	Tract & Year
Observations	19.423	19.423	19.423	19.423	19.423	19.423	19.423	19.423	19.423
R2	0,24	0,24	0,24	0,24	0,24	0,24	0,24	0,24	0,24
BIC	111.362	111.415	111.428	111.425	111.399	111.367	111.378	111.434	111.431

Over-Dispersion	10,43	10,35	10,34	10,35	10,38	10,4	10,44	10,33	10,31

Supplementary Table 5: Negative binomial regression models predicting the number of new roofing permits following a fire treatment - treatment varies with distance. A census tract is classified as treated when its population-weighted distance to a wildfire has been less than a specified cutoff at any point in the past three years. In these specifications, the cutoff point varies between 1 kilometer and 50 kilometers. Other treatment specifications include the number of treated houses. Placebo treatment is defined as the years prior to a wildfire. Dependent variable is the number of roofing permits per census tract, winsorized at the 1st and 99th percentile. Standard errors are clustered as denominated and in parentheses. All control variables are normalized to the [0,1] interval so the coefficients represent the difference in effect size when the corresponding variable is at its maximum (coded as 1) vs. minimum (coded as 0). Statistical significance levels are . p<0.1, * p<0.05, **p<0.01, and ***p<0.001.

Robustness Checks - Treatment definition (2)

	1	2	3	4	5	6	7	8	9
Dependent Variable					*No. of Roofing Permits*				
	Coefficient (clustered standard error; t-statistic; P value)	*Coefficient (clustered standard error; t-statistic; P value)*	*Coefficient (clustered standard error; t-statistic; P value)*	*Coefficient (clustered standard error; t-statistic; P value)*	*Coefficient (clustered standard error; t-statistic; P value)*	*Coefficient (clustered standard error; t-statistic; P value)*	*Coefficient (clustered standard error; t-statistic; P value)*	*Coefficient (clustered standard error; t-statistic; P value)*	*Coefficient (clustered standard error; t-statistic; P value)*
Duration 2 years; Distance 1k	0.14** (0.05; 2.72;0.006)								
Duration 2 years; Distance 2.5k		0.11** (0.04; 2.64;0.008)							
Duration 2 years; Distance 5k			0.11** (0.04; 2.77;0.006)						
Duration 2 years; Distance 10k				0.11** (0.04; 2.65;0.008)					
Duration 2 years; Distance 20k					0.07 (0.05; 1.45;0.148)				
Duration 4 years; Distance 1k						0.21*** (0.04; 5.12;<0.001)			
Duration 4 years; Distance 2.5k							0.17*** (0.04; 4.17;<0.001)		
Duration 4 years; Distance 5k								0.15** (0.05; 3.02;0.003)	
Duration 4 years; Distance 10k									0.13* (0.06; 2.32;0.02)
Duration 4 years; Distance 20k									
Mean Household Income	-0.84 (0.49; -1.7;0.089)	-0.85 (0.49; -1.72;0.085)	-0.85 (0.49; -1.74;0.082)	-0.9 (0.47; -1.9;0.057)	-0.92* (0.46; -2;0.046)	-0.83 (0.5; -1.67;0.094)	-0.85 (0.49; -1.73;0.084)	-0.84 (0.49; -1.73;0.083)	-0.91 (0.48; -1.91;0.056)
WUI (Share)	0.62 (0.81; 0.77;0.442)	0.61 (0.8; 0.76;0.446)	0.6 (0.8; 0.75;0.45)	0.63 (0.78; 0.81;0.419)	0.65 (0.81; 0.8;0.425)	0.64 (0.8; 0.79;0.429)	0.59 (0.79; 0.75;0.455)	0.55 (0.8; 0.68;0.495)	0.49 (0.79; 0.62;0.534)
Installed Base	0.81** (0.28; 2.86;0.004)	0.81** (0.28; 2.84;0.004)	0.8** (0.28; 2.83;0.005)	0.77** (0.28; 2.78;0.005)	0.75** (0.26; 2.87;0.004)	0.81** (0.28; 2.87;0.004)	0.8** (0.28; 2.88;0.004)	0.78** (0.27; 2.89;0.004)	0.75** (0.26; 2.87;0.004)
Moved last 3 years (Share)	0.38 (0.31; 1.24;0.215)	0.38 (0.31; 1.25;0.212)	0.38 (0.3; 1.26;0.208)	0.39 (0.29; 1.36;0.175)	0.39 (0.29; 1.34;0.181)	0.38 (0.31; 1.24;0.216)	0.37 (0.3; 1.25;0.212)	0.37 (0.29; 1.28;0.202)	0.4 (0.28; 1.39;0.163)
Wildfire Damaged Houses	1.83*** (0.52; 3.51;<0.001)	1.84*** (0.54; 3.4;<0.001)	1.78*** (0.54; 3.3;<0.001)	1.82*** (0.55; 3.35;<0.001)	1.92*** (0.54; 3.56;<0.001)	1.85*** (0.51; 3.6;<0.001)	1.86*** (0.52; 3.58;<0.001)	1.86*** (0.51; 3.68;<0.001)	1.86*** (0.52; 3.54;<0.001)
Firewise Communities	0.03* (0.01; 2.16;0.031)	0.03* (0.01; 2.16;0.031)	0.03* (0.01; 2.18;0.029)	0.03* (0.01; 2.18;0.029)	0.03* (0.02; 2.13;0.033)	0.03* (0.01; 2.18;0.03)	0.03* (0.01; 2.2;0.028)	0.03* (0.01; 2.19;0.028)	0.03* (0.01; 2.09;0.037)
Owner Occupied (Share)	0.29 (0.21; 1.33;0.183)	0.29 (0.21; 1.36;0.175)	0.3 (0.21; 1.4;0.162)	0.32 (0.21; 1.51;0.13)	0.31 (0.21; 1.48;0.138)	0.28 (0.21; 1.31;0.19)	0.29 (0.21; 1.36;0.172)	0.31 (0.21; 1.45;0.147)	0.34 (0.21; 1.6;0.109)
Count of Small Fires (Log)	0.33* (0.16; 2.01;0.044)	0.33* (0.16; 2.01;0.044)	0.33* (0.16; 2.01;0.044)	0.33* (0.16; 2.01;0.044)	0.32 (0.16; 1.96;0.05)	0.33* (0.16; 2;0.046)	0.33* (0.17; 2;0.045)	0.33* (0.16; 1.99;0.047)	0.33* (0.16; 1.98;0.047)
Fixed-Effects:									
Census Tract	*Yes*	*Yes*	*Yes*	*Yes*	*Yes*	*Yes*	*Yes*	*Yes*	*Yes*
Year	*Yes*	*Yes*	*Yes*	*Yes*	*Yes*	*Yes*	*Yes*	*Yes*	*Yes*
Model Family	Neg. Binom	Neg. Binom	Neg. Binom	Neg. Binom	Neg. Binom	Neg. Binom	Neg. Binom	Neg. Binom	Neg. Binom
Clustered SE	Tract & Year	Tract & Year	Tract & Year	Tract & Year	Tract & Year	Tract & Year	Tract & Year	Tract & Year	Tract & Year
Observations	19.423	19.423	19.423	19.423	19.423	19.423	19.423	19.423	19.423
R2	0,24	0,24	0,24	0,24	0,24	0,24	0,24	0,24	0,24
BIC	111.430	111.426	111.415	111.387	111.396	111.421	111.412	111.393	111.366
Over-Dispersion	10,34	10,34	10,35	10,38	10,34	10,35	10,37	10,39	10,43

Supplementary Table 6: Negative binomial regression models predicting the number of new roofing permits following a fire treatment - treatment varies by treatment length and distance cutoff. DAC status definition follows the identification by Justice40. treatment varies with distance. A census tract is classified as treated when its population-weighted distance to a wildfire has been less than a cutoff at any point in the past years. Dependent variable is the number of roofing permits per census tract, winsorized at the 1st and 99th percentile. Standard errors are clustered as denominated and in parentheses. All control variables are normalized to the [0,1] interval so the coefficients represent the difference in effect size when the corresponding variable is at its maximum (coded as 1) vs. minimum (coded as 0). Statistical significance levels are . p<0.1, * p<0.05, **p<0.01, and ***p<0.001.

Robustness Checks - DAC vs. non-DAC Treatment

	1	2	3	4	5	6	7	8
Dependent Variable	*No. of Roofing Permits*							
	Coefficient (clustered standard error; t-statistic; P value)	*Coefficient (clustered standard error; t-statistic; P value)*	*Coefficient (clustered standard error; t-statistic; P value)*	*Coefficient (clustered standard error; t-statistic; P value)*	*Coefficient (clustered standard error; t-statistic; P value)*	*Coefficient (clustered standard error; t-statistic; P value)*	*Coefficient (clustered standard error; t-statistic; P value)*	*Coefficient (clustered standard error; t-statistic; P value)*
**DAC Treatment - Original**	0.07 (0.04; 1.76;0.078)							
**non-DAC Treatment - Original**	0.16** (0.05; 2.96;0.003)							
DAC No. Of Houses Treated		-0.1 (0.31; -0.33;0.741)						
non-DAC No. Of Houses Treated		0.7** (0.24; 2.92;0.003)						
DAC Treatment Distance 1k			0.12 (0.22; 0.56;0.573)					
Non-DAC Treatment Distance 1k			0.15*** (0.04; 3.98;<0.001)					
DAC Treatment Distance 2.5k				-0.04 (0.12; -0.35;0.724)				
Non-DAC Treatment Distance 2.5k				0.17*** (0.05; 3.61;<0.001)				
DAC Treatment Distance 5k					0.1 (0.07; 1.53;0.126)			
Non-DAC Treatment Distance 5k					0.16*** (0.05; 3.4;<0.001)			
DAC Treatment Distance 20k						0.08* (0.03; 2.46;0.014)		
Non-DAC Treatment Distance 20k						0.11* (0.05; 2.09;0.037)		
DAC Placebo Treatment								0 (0.02; 0.22;0.827)
Non-DAC Placebo Treatment								0 (0.04; 0.07;0.943)
DAC Treatment Smoke								
Non-DAC Treatment Smoke								
Mean Household Income	-0.94* (0.47; -2.01;0.044)	-0.93* (0.47; -1.97;0.049)	-0.84 (0.49; -1.7;0.09)	-0.87 (0.49; -1.76;0.078)	-0.86 (0.48; -1.79;0.073)	-1.03* (0.48; -2.16;0.031)	-0.47 (0.44; -1.06;0.291)	-0.84 (0.51; -1.66;0.098)
WUI (Share)	0.53 (0.75; 0.71;0.478)	0.45 (0.76; 0.59;0.554)	0.62 (0.81; 0.77;0.443)	0.6 (0.8; 0.74;0.456)	0.56 (0.78; 0.72;0.471)	0.61 (0.8; 0.76;0.447)	0.58 (0.79; 0.74;0.458)	0.61 (0.8; 0.76;0.447)
Installed Base	0.75** (0.27; 2.76;0.006)	0.77** (0.27; 2.81;0.005)	0.81** (0.28; 2.86;0.004)	0.8** (0.28; 2.83;0.005)	0.79** (0.28; 2.84;0.005)	0.72** (0.26; 2.74;0.006)	0.77** (0.27; 2.85;0.004)	0.81** (0.28; 2.92;0.004)
Moved last 3 years (Share)	0.39 (0.29; 1.37;0.17)	0.39 (0.29; 1.34;0.18)	0.38 (0.31; 1.24;0.216)	0.38 (0.3; 1.25;0.21)	0.37 (0.3; 1.26;0.206)	0.39 (0.3; 1.3;0.194)	0.36 (0.28; 1.29;0.198)	0.38 (0.31; 1.23;0.219)
Wildfire Damaged Houses	1.85*** (0.52; 3.58;<0.001)	1.9*** (0.47; 4.04;<0.001)	1.86*** (0.44; 4.22;<0.001)	1.92*** (0.43; 4.45;<0.001)	1.82*** (0.52; 3.53;<0.001)	1.95*** (0.52; 3.75;<0.001)	1.91*** (0.51; 3.76;<0.001)	1.94*** (0.51; 3.83;<0.001)
Firewise Communities	0.03* (0.01; 2.16;0.03)	0.03* (0.01; 1.99;0.047)	0.03* (0.01; 2.16;0.031)	0.03* (0.01; 2.16;0.031)	0.03* (0.01; 2.18;0.029)	0.03* (0.01; 2.17;0.03)	0.03 (0.01; 1.84;0.065)	0.03* (0.02; 1.99;0.047)
Owner Occupied (Share)	0.34 (0.21; 1.64;0.101)	0.34 (0.21; 1.6;0.11)	0.28 (0.21; 1.32;0.186)	0.3 (0.21; 1.39;0.164)	0.31 (0.21; 1.47;0.142)	0.34 (0.21; 1.6;0.109)	0.24 (0.21; 1.11;0.266)	0.28 (0.22; 1.3;0.195)
Count of Small Fires (Log)	0.31 (0.16; 1.93;0.053)	0.3 (0.16; 1.88;0.061)	0.33* (0.16; 2;0.046)	0.33* (0.17; 1.99;0.046)	0.32* (0.16; 1.97;0.049)	0.28 (0.17; 1.69;0.092)	0.37* (0.17; 2.14;0.032)	0.33* (0.16; 2.03;0.042)
Fixed-Effects:								
Census Tract	*Yes*	*Yes*	*Yes*	*Yes*	*Yes*	*Yes*	*Yes*	*Yes*
Year	*Yes*	*Yes*	*Yes*	*Yes*	*Yes*	*Yes*	*Yes*	*Yes*

Model Family	Neg. Binom	Neg. Binom	Neg. Binom	Neg. Binom	Neg. Binom	Neg. Binom	Neg. Binom	Neg. Binom
Clustered SE	Tract & Year	Tract & Year	Tract & Year	Tract & Year	Tract & Year	Tract & Year	Tract & Year	Tract & Year
Observations	19.423	19.423	19.423	19.423	19.423	19.423	19.423	19.423
R2	0,24	0,24	0,24	0,24	0,24	0,24	0,24	0,24
BIC	111.364	111.421	111.438	111.428	111.408	111.375	111.444	111.441
Over-Dispersion	10,45	10,36	10,34	10,36	10,38	10,39	10,33	10,31

Supplementary Table 7: Negative binomial regression models predicting the number of new roofing permits following a fire treatment - treatment is split by DAC status and varies with distance. DAC status definition follows the identification by Justice40. Dependent variable is the number of roofing permits per census tract, winsorized at the 1st and 99th percentile. Standard errors are clustered as denominated and in parentheses.. All control variables are normalized to the [0,1] interval so the coefficients represent the difference in effect size when the corresponding variable is at its maximum (coded as 1) vs. minimum (coded as 0). Statistical significance levels are . p<0.1, * p<0.05, **p<0.01, and ***p<0.001.

Robustness Checks - Definition of Ownership levels

	1	2	3	4
Dependent Variable	*No. of Roofing Permits*			
	Coefficient (clustered standard error; t-statistic; P value)	*Coefficient (clustered standard error; t-statistic; P value)*	*Coefficient (clustered standard error; t-statistic; P value)*	*Coefficient (clustered standard error; t-statistic; P value)*
DAC * Low Ownership (15th)	0.08 (0.09; 0.91;0.365)			
Non-DAC * Low Ownership (15th)	-0.02 (0.15; -0.16;0.871)			
DAC * High Ownership (75th)	0 (0.08; -0.04;0.967)			
Non-DAC * High Ownership (75th)	0.25*** (0.06; 4.05;<0.001)			
DAC * Low Ownership (15th)		0.18 (0.18; 0.96;0.337)		
Non-DAC * Low Ownership (15th)		-0.09 (0.2; -0.47;0.635)		
Non-DAC * High Ownership (85th)		0.3*** (0.08; 3.58;<0.001)		
DAC * Low Ownership (40th)			0.09 (0.06; 1.59;0.113)	
Non-DAC * Low Ownership (40th)			-0.02 (0.09; -0.19;0.853)	
DAC * High Ownership (60th)			0.14* (0.07; 2.11;0.035)	
Non-DAC * High Ownership (60th)			0.23*** (0.06; 4.09;<0.001)	
DAC * Low Ownership (50th)				0.04 (0.05; 0.77;0.44)
Non-DAC * Low Ownership (50th)				0.08 (0.08; 0.99;0.32)
DAC * High Ownership (50th)				0.17* (0.07; 2.47;0.014)
Non-DAC * High Ownership (50th)				0.22*** (0.06; 3.75;<0.001)
Mean Household Income	-0.88 (0.5; -1.77;0.077)	-0.83 (0.5; -1.66;0.098)	-0.92 (0.49; -1.89;0.059)	-0.94 (0.48; -1.94;0.052)
Moved last 3 years (Share)	0.44 (0.27; 1.64;0.1)	0.46 (0.29; 1.59;0.112)	0.43 (0.26; 1.68;0.094)	0.44 (0.26; 1.68;0.092)
Wildfire Damaged Houses	1.93*** (0.5; 3.86;<0.001)	1.94*** (0.51; 3.78;<0.001)	1.8*** (0.47; 3.8;<0.001)	1.8*** (0.52; 3.43;<0.001)
Firewise Communities	0.03 (0.01; 1.89;0.059)	0.03 (0.01; 1.84;0.066)	0.03* (0.01; 2.01;0.045)	0.03 (0.02; 1.87;0.062)
Count of Small Fires (Log)	0.33 (0.17; 1.93;0.053)	0.34* (0.17; 1.98;0.047)	0.33 (0.17; 1.9;0.057)	0.32 (0.17; 1.93;0.054)
Fixed-Effects:				
Census Tract	*Yes*	*Yes*	*Yes*	*Yes*
Year	*Yes*	*Yes*	*Yes*	*Yes*
Model Family	Neg. Binom	Neg. Binom	Neg. Binom	Neg. Binom
Clustered SE	Tract & Year	Tract & Year	Tract & Year	Tract & Year
Observations	21,688	21,688	21,688	21,688
R2	0.24	0.24	0.24	0.24
BIC	128,065	128,063	128,026	128,017
Over-Dispersion	8.40	8.38	8.42	8.43

Supplementary Table 8: Negative binomial regression models predicting the number of new roofing permits following a fire treatment - treatment is split by

DAC status and ownership level. DAC status definition follows the identification by Justice40. Low ownership is defined in our main specification as less than the 25th percentile of owner occupied houses and high ownership as above the 75th percentile. In these specifications, we vary the cutoff points as indicated.Dependent variable is the number of roofing permits per census tract, winsorized at the 1st and 99th percentile. Standard errors are clustered as denominated and in parentheses.. All control variables are normalized to the [0,1] interval so the coefficients represent the difference in effect size when the corresponding variable is at its maximum (coded as 1) vs. minimum (coded as 0). Statistical significance levels are . p<0.1, * p<0.05, **p<0.01, and ***p<0.001.

Robustness Checks - Model robustness

	1	2	3
Dependent Variable		*No. of Roofing Permits*	
	Coefficient (clustered standard error; t-statistic; P value)	*Coefficient (clustered standard error; t-statistic; P value)*	*Coefficient (clustered standard error; t-statistic; P value)*
DAC Treated	0.09* (0.04; 2.25;0.024)	0.07** (0.03; 2.57;0.005)	0.07 (0.3; 0.23;0.825)
non-DAC Treated	0.19** (0.06; 3.28;0.001)	0.18*** (0.02; 10.46;<0.001)	1.66** (0.43; 3.85;0.005)
Mean Household Income	-0.95 (0.5; -1.9;0.057)	-0.97*** (0.2; -4.84;<0.001)	0.74 (2.75; 0.27;0.794)
WUI (Share)	0.76 (0.73; 1.05;0.294)	0.49 (0.42; 1.15;0.125)	2.19 (5.77; 0.38;0.714)
Moved last 3 years (Share)	0.5 (0.28; 1.79;0.073)	0.42*** (0.12; 3.51;<0.001)	2.47 (1.3; 1.89;0.095)
Wildfire Damaged Houses	1.84*** (0.51; 3.58;<0.001)	1.55*** (0.4; 3.85;<0.001)	7.53 (3.58; 2.1;0.068)
Firewise Communities	0.03 (0.02; 1.73;0.084)	0.02* (0.01; 1.75;0.04)	0.74** (0.22; 3.42;0.009)
Owner Occupied (Share)	0.33 (0.21; 1.54;0.124)	0.29** (0.11; 2.63;0.004)	0.58 (1.14; 0.51;0.624)
Count of Small Fires (Log)	0.32* (0.16; 1.96;0.049)	0.21*** (0.06; 3.76;<0.001)	1.1 (0.9; 1.22;0.256)
Fixed-Effects:			
Census Tract	*Yes*	*Yes*	*Yes*
Year	*Yes*	*Yes*	*Yes*
Model Family	Neg. Binom	Poisson	OLS
Clustered SE	Tract & Year	Tract & Year	Tract & Year
Observations	21,688	22,15	22,15
R2	0.24	0.21	0.21
BIC	128,026	154,187	154,187
Over-Dispersion	8.42	--	--

Supplementary Table 9: Comparison of negative binomial, Poisson, and ordinary least squares regression models predicting the number of new roofing permits following a fire treatment - treatment is split by DAC status. DAC status definition follows the identification by Justice40. Dependent variable is the number of roofing permits per census tract, winsorized at the 1st and 99th percentile. Standard errors are clustered as denominated and in parentheses. All control variables are normalized to the [0,1] interval so the coefficients represent the difference in effect size when the corresponding variable is at its maximum (coded as 1) vs. minimum (coded as 0). Statistical significance levels are . p<0.1, * p<0.05, **p<0.01, and ***p<0.001.

Panel Regression for number of roofing permits

	1	2	3	4	5
Dependent Variable			*No. of Roofing Permits*		
	Coefficient (clustered standard error; t-statistic; P value)	*Coefficient (clustered standard error; t-statistic; P value)*	*Coefficient (clustered standard error; t-statistic; P value)*	*Coefficient (clustered standard error; t-statistic; P value)*	*Coefficient (clustered standard error; t-statistic; P value)*
DAC Indicator	-0.61*** (0.09; -6.54;<0.001)	-0.3*** (0.06; -5.29;<0.001)	-0.19** (0.07; -2.89;0.004)	-0.18** (0.06; -3.06;0.002)	-0.25*** (0.04; -6.72;<0.001)
Median Income		1.87*** (0.43; 4.37;<0.001)	0.62* (0.27; 2.27;0.023)	0.71* (0.34; 2.08;0.037)	0.43 (0.44; 0.96;0.335)
Moved more than 10 years ago (Share)			0.83** (0.31; 2.67;0.008)	0.84** (0.27; 3.12;0.002)	0.26 (0.31; 0.84;0.401)
GINI			0.82* (0.32; 2.54;0.011)	0.75** (0.27; 2.73;0.006)	0.89*** (0.24; 3.78;<0.001)
No. of Buildings (Thousands)			0.29* (0.12; 2.48;0.013)	0.32* (0.16; 2.08;0.038)	0.34* (0.15; 2.25;0.025)
Fire Risk			-0.19* (0.09; -2.05;0.041)	-0.18* (0.08; -2.27;0.023)	-0.18* (0.08; -2.14;0.032)
Mobile Homes (Share)			-0.73*** (0.18; -4.09;<0.001)	-0.81*** (0.16; -5.14;<0.001)	-0.85*** (0.15; -5.57;<0.001)
Owner Occupied (Share)			0.75 (0.42; 1.79;0.074)	0.79 (0.41; 1.93;0.054)	1.01* (0.41; 2.5;0.013)
Built after 2010 (Share)			0.61 (0.51; 1.21;0.226)	0.67 (0.44; 1.55;0.122)	0.02 (0.38; 0.05;0.959)
Built before 1990 (Share)			1.15** (0.42; 2.75;0.006)	1.08* (0.42; 2.57;0.01)	1.07** (0.41; 2.62;0.009)
Insurance Coverage				0.15 (0.4; 0.36;0.718)	0.11 (0.56; 0.2;0.843)
Residential Solar				-0.28 (0.29; -0.96;0.338)	-0.33 (0.28; -1.15;0.251)
Fixed-Effects:					
County	*Yes*	*Yes*	*Yes*	*Yes*	*Yes*
Year	*No*	*No*	*No*	*No*	*Yes*
Model Family	Neg. Binom	Neg. Binom	Neg. Binom	Neg. Binom	Neg. Binom
Clustered SE	County	County	County	County	County & Year
Observations	21,996	21,996	21,996	21,996	21,996
R2	0.04	0.04	0.06	0.06	0.07
BIC	132,587	131,818	129,245	129,159	128,767
Over-Dispersion	0.96	01.01	1.18	1.18	1.22

Supplementary Table 10: Negative binomial panel regression predicting the number of roofing permits in a census tract. Dependent variable is the number of roofing permits per census tract, winsorized at the 1st and 99th percentile. Standard errors are clustered as denominated and in parentheses. All control variables are normalized to the [0,1] interval so the coefficients represent the difference in effect size when the corresponding variable is at its maximum (coded as 1) vs. minimum (coded as 0). Statistical significance levels are . p<0.1, * p<0.05, **p<0.01, and ***p<0.001.

Supplementary Note 5: Roofing Benefits

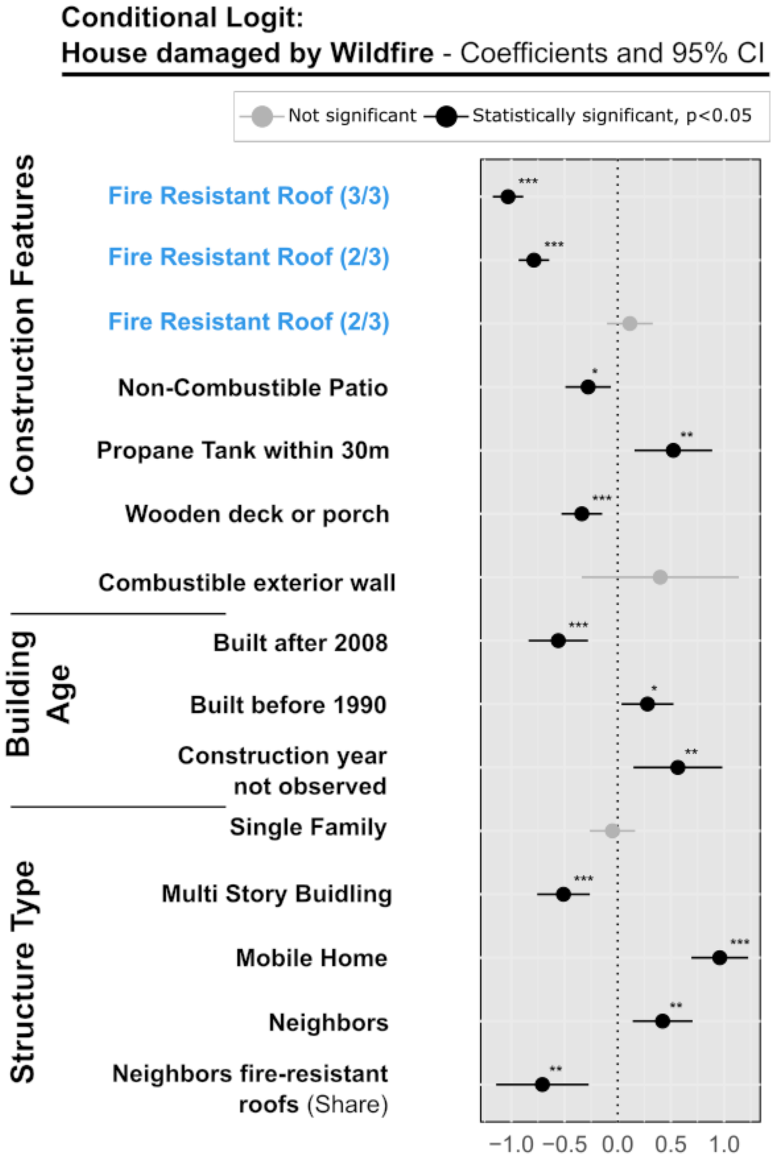

Supplementary Figure 2: Conditional logit regression model that quantifies the association between fire-resistant roofs and the probability of a building being damaged by a wildfire conditional on wildfire exposure**.** Displayed values are the model coefficients, i.e., negative values signify a decrease in damage probability. Fire-resistant roof criteria measures if three criteria are fulfilled: Roof Cover (Asphalte, Metal, Concrete or Tiles), Vents (Mesh Screens > ⅛) and Eaves (No eaves or enclosed eaves). Model includes Fire-Event fixed effects. The sample includes n = 60,986 houses 2013 and 2021 from California that were exposed to wildfires. All independent variables are normalized. Statistical significance levels are * $p<0.05$, ** $p<0.01$, and *** $p<0.001$.

	1	2	3	4
Dependent Variable		*House damaged in wildfire (1 or 0)*		
	Coefficient (clustered standard error; t-statistic; P value)	*Coefficient (clustered standard error; t-statistic; P value)*	*Coefficient (clustered standard error; t-statistic; P value)*	*Coefficient (clustered standard error; t-statistic; P value)*
3/3 Fire Resistant Roof Criteria	-1.34*** (0.19; -6.91;<0.001)	-1.36*** (0.14; -10.04;<0.001)		
2/3 Fire Resistant Roof Criteria	-0.74*** (0.11; -6.93;<0.001)	-0.77*** (0.09; -8.58;<0.001)		
1/3 Fire Resistant Roof Criteria	0.11 (0.2; 0.54;0.59)	0.06 (0.12; 0.45;0.651)		
Vent Criteria			-0.41** (0.13; -3.24;0.001)	-0.46*** (0.11; -4.26;<0.001)
Roof Cover Criteria			-0.43 (0.23; -1.86;0.063)	-0.39** (0.14; -2.84;0.004)
Vent Criteria			-1.17*** (0.08; -14.66;<0.001)	-1.3*** (0.08; -17.14;<0.001)
Num Neighbors		0.38 (0.22; 1.73;0.084)		0.36 (0.22; 1.66;0.096)
Mobile Home		1.91*** (0.19; 10.21;<0.001)		2.11*** (0.2; 10.61;<0.001)
Multi Story Building		-0.48*** (0.13; -3.61;<0.001)		-0.45*** (0.14; -3.3;<0.001)
Single Family Home		0.77*** (0.13; 5.98;<0.001)		0.79*** (0.14; 5.71;<0.001)
Age not observed		0.7** (0.24; 2.93;0.003)		0.69** (0.26; 2.67;0.008)
Constructed before 1990		0.26 (0.15; 1.75;0.081)		0.21 (0.15; 1.42;0.155)
Constructed after 2008		-0.69*** (0.12; -5.9;<0.001)		-0.65*** (0.13; -5.02;<0.001)
Combustible exterior wall		0.3 (0.33; 0.91;0.36)		0.22 (0.34; 0.65;0.516)
Wooden Porch		-0.39** (0.13; -3.11;0.002)		-0.42*** (0.12; -3.44;<0.001)
Non-combustible patio		-0.25 (0.16; -1.59;0.111)		-0.23 (0.17; -1.4;0.162)
Propan tank within 30m		0.59** (0.21; 2.79;0.005)		0.55** (0.21; 2.66;0.008)
Fixed-Effects:				
Fire Event	Yes	Yes	Yes	Yes
Model Family	Cond. Logit	Cond. Logit	Cond. Logit	Cond. Logit
Observations	40,673	40,673	40,673	40,673
R2	0.57	0.54	0.58	0.55
Wald Test	1	0	1	0
LR Test	26215.10	27848.80	25946.30	27611.30

Supplementary Table 11: Conditional logit regression models predicting the risk reduction benefits of fire-resistant roofs. Displayed values are the coefficients of a conditional logit regression, i.e. values below 0 signify a decrease in risk and values above 0 an increase in risk. Standard errors are in parentheses. Fire-resistant roof criteria measures how many out of three criteria are fulfilled: Roof Cover (Asphalte, Metal, Concrete or Tiles), Vents (Mesh Screens > ⅛) and Eaves (No eaves or enclosed eaves). Statistical significance levels are * p<0.05, **p<0.01, and ***p<0.001.

	1	2	3	4	5
Fires Excluded	*Top 1*	*Top 1-2*	*Top 1-3*	*Top 1-4*	*Top 1-5*
Dependent Variable	*House damaged in wildfire (1 or 0)*				
3/3 Fire Resistant Roof Criteria	-1.5*** (0.15)	-1.5*** (0.19)	-1.6*** (0.23)	-1.7*** (0.29)	-2.1*** (0.25)
2/3 Fire Resistant Roof Criteria	-0.84*** (0.10)	-0.86*** (0.15)	-0.94*** (0.17)	-1.1*** (0.21)	-1.4*** (0.26)
1/3 Fire Resistant Roof Criteria	0.03 (0.11)	-0.08 (0.19)	-0.17 (0.21)	-0.23 (0.25)	-0.44 (0.33)
Num Neighbors	0.28 (0.24)	0.03 (0.29)	0.006 (0.31)	0.03 (0.33)	0.34. (0.19)
Mobile Home	2.0*** (0.22)	2.1*** (0.32)	2.1*** (0.35)	2.0*** (0.43)	1.8*** (0.35)
Multi Story Building	-0.49** (0.15)	-0.62*** (0.14)	-0.67*** (0.16)	-0.79*** (0.16)	-0.65** (0.22)
Single Family Home	0.87*** (0.12)	0.84*** (0.24)	0.85** (0.26)	0.85* (0.33)	0.59** (0.22)
Age not observed	0.64* (0.26)	0.24 (0.16)	0.24 (0.21)	0.28 (0.18)	0.20 (0.16)
Constructed before 1990	0.08 (0.11)	0.12 (0.14)	0.21 (0.17)	0.30* (0.13)	0.21* (0.11)
Constructed after 2008	-0.75*** (0.15)	-0.71*** (0.18)	-0.60*** (0.14)	-0.50*** (0.15)	-0.52*** (0.15)
Combustible exterior wall	-0.14 (0.11)				
Wooden Porch	-0.49*** (0.11)	-0.49*** (0.11)	-0.54*** (0.11)	-0.60*** (0.13)	-0.45*** (0.09)
Non-combustible patio	-0.09 (0.17)	-0.07 (0.18)	-0.008 (0.21)	0.18 (0.19)	0.13 (0.18)
Propan tank within 30m	0.63** (0.22)	0.59** (0.20)	0.62** (0.22)	0.69** (0.24)	0.33*** (0.07)
Fixed-Effects:					
Fire Event	Yes	Yes	Yes	Yes	Yes
Model Family	Cond. Logit	Cond. Logit	Cond. Logit	Cond. Logit	Cond. Logit
Observations	25,481	18,713	16,596	13,927	10,670
Squared Observations	0.49	0.37	0.34	0.38	0.43
R2	0.42	0.33	0.30	0.33	0.38
BIC	20919.1	16799.6	14552	11783.9	8743.8

Supplementary Table 12: Conditional logit regression models predicting the risk reduction benefits of fire-resistant roofs, gradually excluding the five most destructive fires. Gradual exclusion of the five most destructive fires, including Camp Fire (15,192 observations), Tubbs Fire (6,768 observations) and LNU & CZU Lightning Complex (2,117 observations). Displayed values are the exponentiated coefficients of a conditional logit regression, i.e. values below 1 signify a decrease in risk and values above 1 an increase in risk. Standard errors are in parentheses. Fire-resistant roof criteria measures how many out of three criteria are fulfilled: Roof Cover (Asphalte, Metal, Concrete or Tiles), Vents (Mesh Screens > ⅛) and Eaves (No eaves or enclosed eaves). Statistical significance levels are * p<0.05, **p<0.01, and ***p<0.001.

Supplementary Note 6: Exposure projection by States

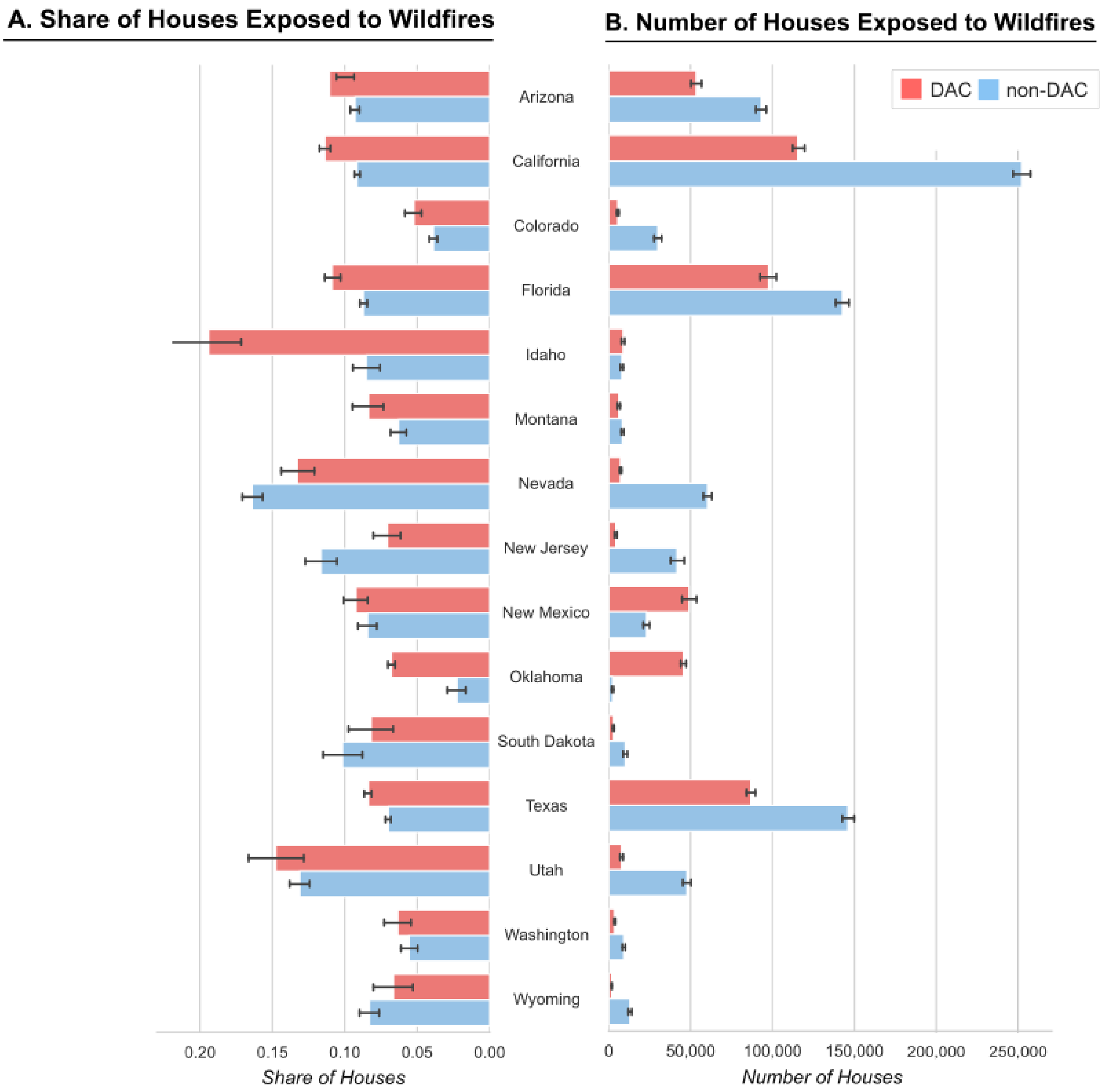

Supplementary Figure 3: Results of exposure projections by states and DAC Status for the next 30 years. A, Share of houses exposed to wildfire for the top 15 most exposed U.S. states as measured by the number of houses exposed. Results from a simulation of the wildfire exposure of the next 30 years across the U.S. with 1,000 repetitions. Exposure does not directly translate to being destroyed by wildfire. The sample includes $n = 27{,}436$ census tracts. **B**, Number of houses exposed to wildfire. The sample includes $n = 27{,}436$ census tracts.

Supplementary Note 7: Firewise Communities analysis

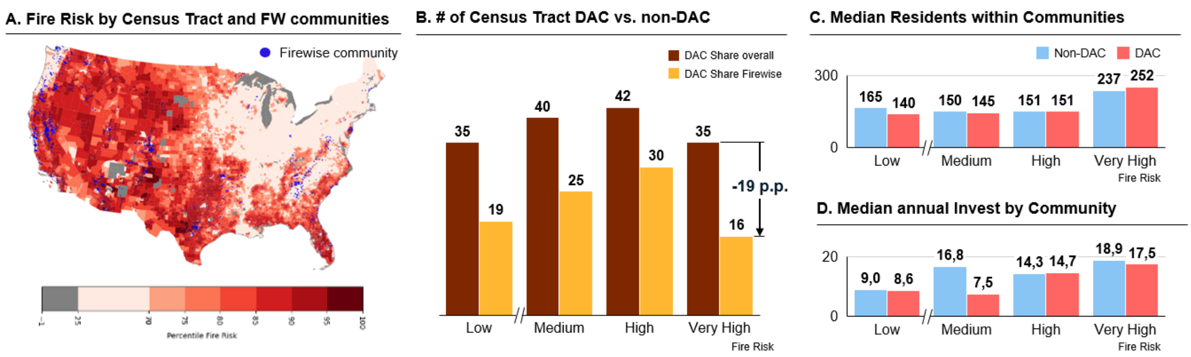

Supplementary Figure 4: Firewise communities within the US. A, Location of Firewise communities which are considered as "In Good Standing" as of 2022 and Fire Risk by Percentiles within the US. The sample includes n= 2,076 Firewise communities. **B**, Comparison of share of census tracts which are considered disadvantaged and share of firewise communities located in disadvantaged communities split by fire risk percentiles. The sample includes n= 2,076 Firewise communities. **C**, Median number of residents within Firewise communities split by fire risk percentiles. The sample includes n= 2,076 Firewise communities. **D**, Median annual investment by Firewise communities which are considered as "In Good Standing" as of 2022. Calculated as the "Total Invest" divided by years since establishment. The sample includes n= 2,076 Firewise communities.

Firewise is a voluntary program by the National Fire Protection Association (NFPA) that encourages local solutions for safety by involving homeowners which can unite and form a "Firewise community". The Firewise program, while being one among several wildfire prevention initiatives in the U.S., is particularly notable due to its prominence and the fact that it acts as a robust proxy for vegetation management and wildfire risk awareness. For our analysis, we utilized data on Firewise communities "In Good Standing" as of 2022 across the entire U.S. As depicted in Supplementary Fig. 4A, there is a substantial state-wise clustering of these communities, with California, in particular, having 597 communities. This can be attributed to earlier state support for the program, and potentially, the role of peer effects in their establishment.

However, our analysis revealed a significant underrepresentation of Firewise communities within DAC tracts across all levels of risk, consistent with prior, but geographically more limited research[17,19]. Our analysis shows an especially notable disparity between the proportion of disadvantaged census tracts within the highest fire risk category and their representation in Firewise communities. Despite the fact that 35% of tracts in the highest fire risk category are disadvantaged, only 16% of Firewise communities within this same risk category are within DACs (Supplementary Fig. 4B). Also in terms of peer effects, we observe stronger clustering for non-DAC communities: Non-DAC Firewise communities within very high wildfire risk areas have on average 94 other firewise communities within 100km radius, whereas DAC Firewise communities have only 63 others in the same radius. This underscores the existence

of a clear gap in participation in such preventive programs among DACs, suggesting deeper inequity in wildfire preparedness and resilience.

Even though DACs are underrepresented in these prevention programs, there are indications that once DACs engage in fire prevention, they are equally involved. For example, there is no meaningful difference in their average years of existence or in median investments towards fire prevention (Supplementary Fig. 4C). The median investment is indeed lower for DACs, but not substantially so. Investment in the context of the firewise communities can be financial or a time investment where each voluntary hour is priced according to fixed criteria. The distribution of investment is skewed, with the mean investment being higher for non-DACs. However, this difference in mean investment is less pronounced in the highest risk category. Per-member investment is substantially smaller within DAC communities, but this is counterbalanced by the larger size of DAC Firewise communities, with an average size of 821 vs. 953, and median sizes of 252 vs. 237, respectively. Furthermore, it is essential to acknowledge that given the median income of DAC areas, the contributions in DAC Firewise communities represent a substantial proportion of their income. Specifically, the median investment per community member and year of $53 corresponds to 0.1 % of the median income in a disadvantaged tract, whereas the $67 within a non-DAC Firewise community corresponds to only 0.07% of the median income in a non-DAC tract (in California the 2020 median income for disadvantaged tracts was $52,353 and $94,597 in a non-disadvantaged tracts). These findings suggest that despite facing resource constraints, DACs show significant levels of community involvement in wildfire prevention initiatives.

Supplementary Note 8: Sample Overview

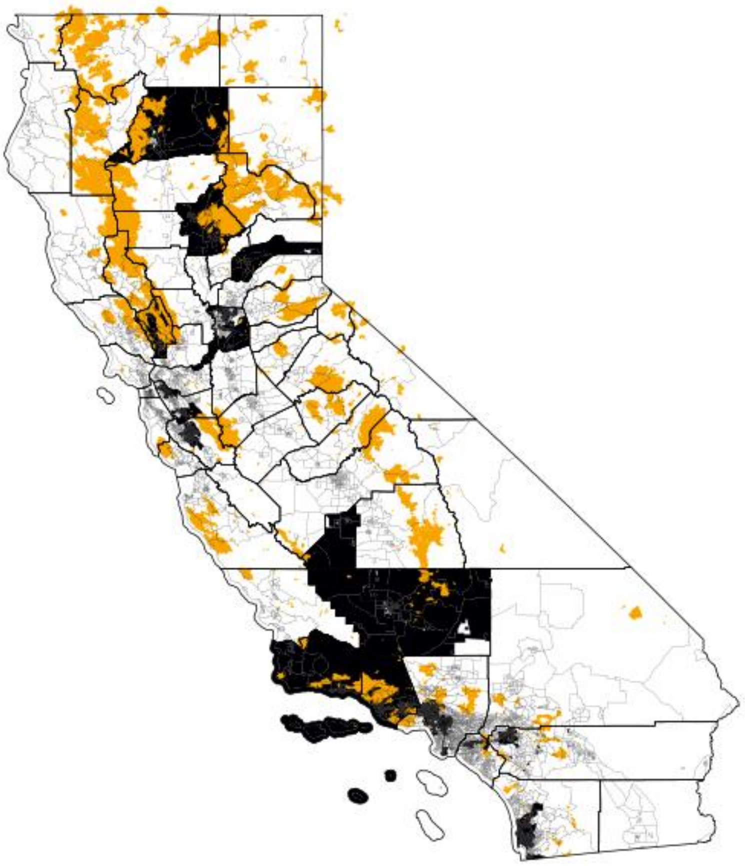

Supplementary Figure 5: Sample overview of building permit database for California and wildfires. Overview of the Fires from 2013 - 2021 in California in orange and the census tracts with roofing permits in black which are used. The sample includes $n = 2{,}563$ census tracts. Fire perimeters were extracted using CAL FIRE's fire repository (https://frap.fire.ca.gov/mapping/gis-data/). Tract boundaries were obtained from the United States Census Bureau[51]. See the following link for the location of the original vector data: https://www.census.gov/cgi-bin/geo/shapefiles/index.php

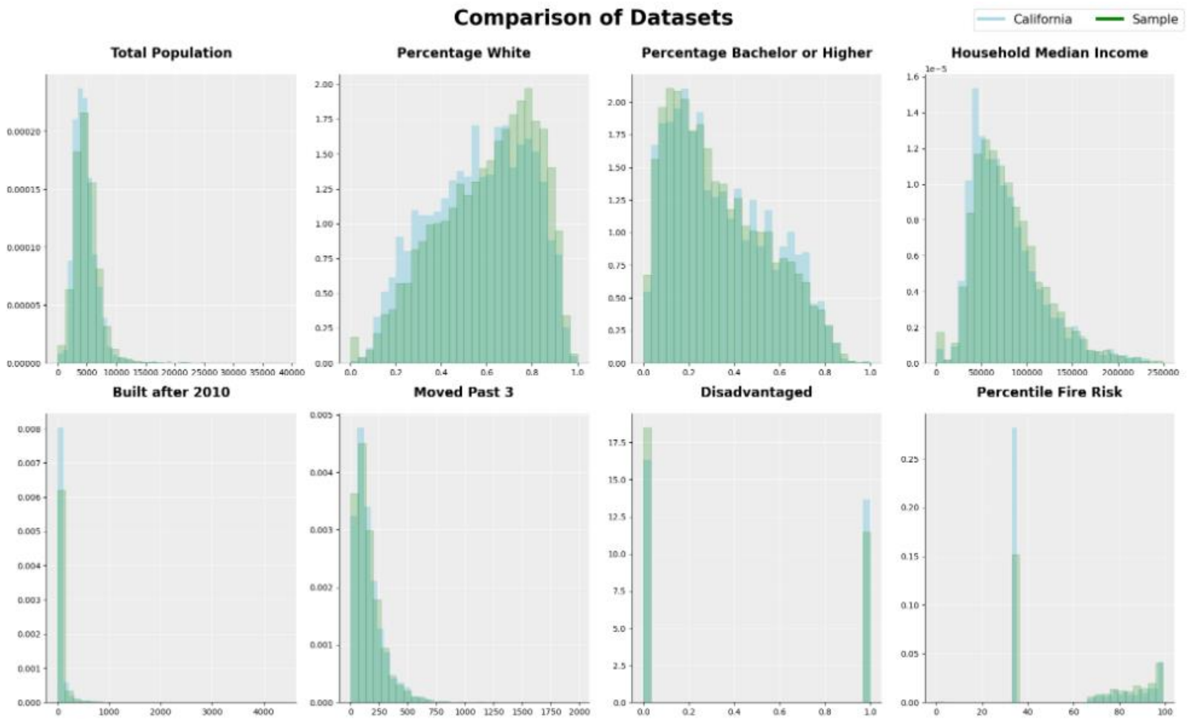

Supplementary Figure 6: Density plots of control variables to compare California and sample in use. ACS information from 2019 used for the variables Total Population, Percentage White, Percentage Bachelor or Higher, Household Median Income, Built after 2010 and Moved in the past three years. For the variables “Disadvantaged” and “Percentile Fire Risk” data is from the Justice40 initiative dataset. Plots show high congruence and high validity of our sample. Only major difference is with respect to percentile fire risk as we naturally focused on potentially fire affected census tracts in our search. The sample includes *n = 2,563* census tracts and overall in California we observed *n=8,046* census tracts.

Supplementary Note 9: Dataset Construction (Permits Database and Measuring Distance to Fire)

Collecting and geo-locating roofing permits. We constructed a panel dataset covering roughly 2,563 census tracts for 9 years from 2013 to 2021 using two principal data sources: (1) building permits and (2) assessor parcel maps. Roofing permits can be obtained from city authorities - typically the Department of Building and Planning. We have identified the responsible offices and personnel in all California counties and contacted each of them. Consequently, we were able to collect more building permits than were publicly available online. We analyzed and geolocated permits from 16 counties across California. Some additional permit databases were identified but were discarded due to incomplete databases (e.g., no full addresses or APNs available), or if matching was not feasible (e.g., if the assessor's office does not openly provide parcel maps). Especially in smaller counties, either no digitized form of building permits is available (e.g., Alpine County) or only scans are provided which rendered a structured analysis nearly impossible. While not all permit databases provide exhaustive coverage for an entire county, they offer a reasonably comprehensive representation of the major cities and in several cases also the unincorporated places. To be useful in our context, all permits utilized in this study must at least encompass the address or preferably the parcel number, job description (i.e., the description for which the permit was issued), a distinction between residential and commercial permits, and an indication of the year. We considered all types of permit statuses (e.g. applied vs. issued); however, we excluded commercial roofing renewals. Based on work descriptions, we identified roofing renewals, ensuring that other roof-related permits, such as roof-mounted solar installations, are not included. We avoided double counting by excluding roofing permits for houses that have already applied for a roofing permit in the past year, which might occur if, for example, two permits are registered in the same database for the same year and the same parcel but for the garage and the house.

Using APNs or, in some instances, addresses, we matched the permits to specific house locations using the parcel data obtained from the local assessor’s offices. We then aggregated the roofing permits on the census tract level. During the matching process, depending on the city database's accuracy, approximately 10-30 % of permits were naturally lost as they could not be matched to a parcel and therefore to a precise location. Still, as long as no bias exists within a census tract, our analysis remains valid since we later used census tract fixed effects to account for differences across tracts. To preclude the mere identification of rebuilding effects post-fires, we eliminated houses from our dataset that have been damaged utilizing the DINS database and a 50-meter perimeter surrounding known damaged houses. In an alternative approach, we did not exclude the damaged houses but simply incorporated a count of damaged houses per year into the regression, which did not influence the regression results – indeed, the coefficient was as expected larger.

Measuring distance to fire and smoke exposure. We were using CAL FIRE's fire perimeters repository (https://frap.fire.ca.gov/mapping/gis-data/; Accessed on 21.02.2023) to locate the most extensive fires exceeding 300 acres. For each house (or in some specifications tracts)

location, we determined the nearest "large" fire per year, subsequently computing the geographic distance. We then averaged this distance across all houses within a census tract. This methodology yields a more significant distance metric compared to determining the distance between census tracts and fires, as population distribution across all tracts is not uniform. Note, that given how we construct our treatment variable, our panel includes data on large fires for the years 2011 - 2021 - i.e. two years before the remainder of our data starts. In some robustness checks we focused only on “damaging” fires by using CAL FIRE's Damage Inspection Database (DINS). A fire is designated as "Damaging" if we were able to spatially match at least one impacted house (*“Affected 1-9%”*) within the DINS database to a fire perimeter. Smoke exposure is defined as days with $PM_{2.5}$ concentration attributable to wildfire smoke, relying on the publicly available database from Childs et al.[52]. Depending on the specifications and context, we either summed the smoke exposure by tract and time (either year or week) or we looked at the days with extreme smoke exposure, defined as above the 80th percentile of smoke concentration.

Supplementary Note 10: Identification of a treatment effect

The identification of a treatment effect requires several assumptions. First, it assumes a common trend between the treatment and control groups. To evaluate the dynamics before the treatment in our study, which has different treatment timings, we carried out an event study type of regression, following previous literature[53]. We constructed a tract-specific new timeline relative to the occurrence of a large fire with the help of the indicator variable T_{it}. I_i indicates whether a tract indeed was exposed to a fire.

$$1)\quad y_{it} = \alpha + I_i * \sum_{y=-4}^{4} \beta_y T_{it} + \gamma x_{it} + \delta_{rt} + c_i + \varepsilon_{it}$$

If roofing renewals exhibited similar trends for both treatment and non-treatment tracts prior to a fire, we would expect coefficients that are not statistically different from zero for the years leading up to the fire, and positive, statistically significant coefficients after the fire. A secondary challenge to identification arises from time-variant tract-specific unobservables or other omitted variables, which could result in biased estimates. To address this issue, we conducted a series of robustness checks to eliminate various alternative explanations. For instance, a newly elected county government might promote increased investments in wildfire prevention or implement new regulations. Consequently, we incorporated county-year fixed effects to account for unobserved differences in a county over time, such as regulatory changes or governmental shifts that affect only one county. Another potential concern involves changes in exposure to wildland and combustible fuels. We contend that this change in exposure can be captured by employing the USGS's WUI estimates and interpolating them on an annual basis. Furthermore, one could argue that the roofing decision was made independently from the occurrence of fires. As a consequence, we included, for instance, controls for building characteristics from the ACS, such as building age and duration of residence of the tract inhabitants. Based on previous roofing permits and building age, we also estimated an "adoption potential" to account for differences in the installed base. Moreover, we controlled for varying levels of fire risk, which might also affect the roofing decision. As updates to official wildfire risk maps are not made annually, we used the number of small fires under 300 acres as a proxy for changing fire risk and include it in our regression. Third, to validate a difference-in-difference design, one must eliminate the possibility of fire affecting pre-treatment outcomes and reverse causality. It seems implausible that new roofs could cause a fire; however, one could argue that if properties in a given tract undergo frequent re-roofing, this might indicate to the fire department that these properties are well-maintained and less likely to be at risk of fire, potentially influencing the fire department's response distance. Therefore, we implemented a placebo treatment prior to the fire, yielding statistically insignificant coefficients. Furthermore, a period without fires could suggest to residents that their risk has increased due to the accumulation of burnable fuels. However, this would apply also to all non-treated tracts. Measurement error constitutes another potential threat, implying that in our context, some permits may not be accurately captured, codified, or dated by the respective agencies. Although we cannot rule out the possibility of erroneous permits due to human error, we also have no reason to suspect substantial bias in a specific direction.

Furthermore, one might argue that some permits could have been applied for retroactively, but this is likely a negligible minority as, for instance, the Los Angeles Building Code imposes fines for late permit applications, and appraisers would likely notice a new roof without the necessary permit.

Our difference-in-difference design presents the average treatment effect, while the actual effect of distance to fire on re-roofings per tract may differ across various distances and property types. We proactively leveraged this probable treatment effect heterogeneity, exploring differences across subsets such as disadvantaged communities.

Supplementary Note 11: Extended Methods for Counterfactual Simulation

We conducted counterfactual simulations to assess the impact of roof renewal rates on the future equity gap in wildfire-induced destruction of residential buildings. Specifically, we simulated the expected number of residential buildings that will be destroyed by wildfires over the next 30 years for each census tract in California. We distinguished between different scenarios for wildfire risk, for the risk reduction potential associated with a new roof, and for the roof replacement strategies, which influenced the prioritization order for roof renewals. Our calculations are based on the publicly available dataset of First Street Foundation[54], which indicates the number of properties per wildfire exposure category for all census tracts. This dataset is also used by the U.S. government to inform their assignment of disadvantaged communities[30].

We first simulated the number of new roofs within each tract over a 30-year timeframe with current roof renewal rates. To project the number of new roofs in disadvantaged census tracts, we fitted an exponential function to the number of roof renewals per property from the most recent two years (2020 and 2021) in our database. These years were chosen to evaluate the impacts, if the current roof renewal disparities persist. The procedure was done separately for all disadvantaged and non-disadvantaged communities to replicate the difference in roofing rates. Per run, we drew from these distributions 30 times per tract to simulate 30 years of activity and calculate the number of new roofs by multiplying the result with the number of existing properties, assuming a constant number of buildings within each census tract over time. This assumption is generally supported by the California Department of Finance's projection of a modest 1.3% population growth until 2050[55]. In each census tract, the dataset of First Street Foundation indicates the number of properties within ten categories based on their wildfire risk. In this step of the simulation, we assumed that new roofs would be spread out among these categories, in proportion to their current sizes.

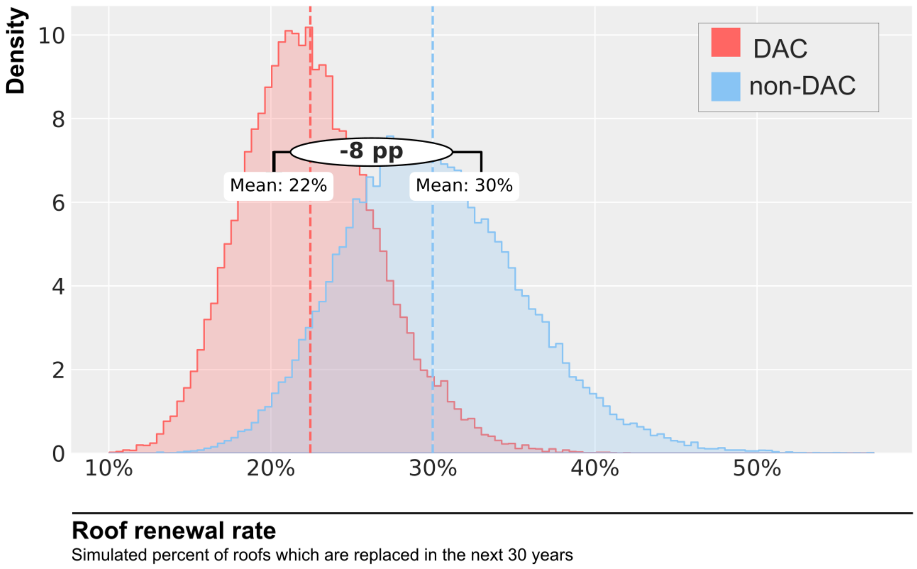

Supplementary Figure 7: Roof renewal rates of DAC vs. non-DAC regions within California. The roof renewal rate is defined as the simulated percentage of properties that get a new roof within 30 years. The roof permits per property for the years 2020 and 2021

were first approximated with a geometric distribution and then 30 random draws per census tract simulate the roof renewal rate in California.

We then gradually increased the number of roof renewals per tract until all houses are considered to have a new roof. To understand the impact of different renewal strategies, we employed three distinct approaches for distributing the new roofs: First, we spread the new roofs uniformly across all ten risk categories in each tract ("uniform"). Every category received an equal proportion of new roofs, ensuring a balanced renewal across varying risk levels. Second, the “highest risk within tract” strategy prioritized roof renewals for buildings in the highest risk categories within each individual census tract before moving to lower-risk categories. Last, the “highest risk across tracts” strategy focused renewal efforts on the highest-risk categories across all tracts, addressing the most vulnerable homes on a broader scale. To analyze these strategies efficiently, we segmented the renewal process into ten stages. For example, in the “uniform” strategy, we used the roof renewal rates of 10%, 20%, up to 100%.

Our simulations incorporated three wildfire risk scenarios, reflecting the range of exposure risks over the next 30 years as estimated by the First Street Foundation. The minimum risk scenario considered the lowest projected risk for each census tract (e.g., 0% for tracts with a 0-1% risk range), while the maximum risk scenario took the highest projected risk (e.g., 1% for tracts with a 0-1% risk range) and the average risk scenario uses the mean wildfire exposure estimates. Within each risk scenario, we further delineated three sub-scenarios based on the potential risk reduction benefits of roof renewal, which we refer to as roof benefit scenarios. To account for the range of possible benefit values, we used 15%, 25% and 35% reduction in likelihood of destruction given wildfire exposure (see Methods on benefits of a roof and Supplementary Note 5). For each of the 27 scenarios (combining the three risk, the three roof benefit cases and the three roof renewal strategies) and 10 roof renewal rates per strategy, we conducted 1,000 individual simulation runs, i.e., in total each tract was sampled 270,000 times, by following the steps outlined below:

1. *Simulating the risk of exposure to a wildfire over the next 30 years*. To simulate the exposure of a census tract to wildfires over the next 30 years, we based our calculations on the wildfire risk estimates from First Street Foundation. As aforementioned, in each census tract, properties are classified into ten risk categories based on their likelihood of exposure to a wildfire (e.g., 0-1%, (Supplementary Figure 8A)).

A. Wildfire Exposure of Tracts

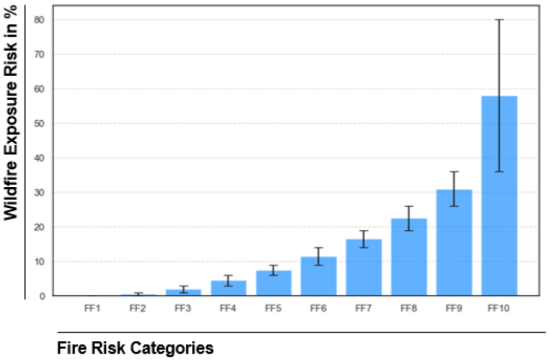

B. Wildfire Exposure Risk adjusted for ignition dependence (Average Risk Scenario)

Category	**Exposure Risk** (First Street Foundation)	Higher category does not burn	Higher category does burn
FF10	58.0%		
FF9	31.0%	11.3%	45.3%
FF8	22.5%	11.0%	46.6%
FF7	16.5%	9.9%	39.4%
FF6	11.5%	7.7%	30.8%
FF5	7.5%	5.6%	22.3%
FF4	4.5%	3.7%	14.7%
FF3	2.0%	1.8%	7.0%
FF2	0.5%	0.5%	1.9%
FF1	0.0%	0.0%	0.0%

Supplementary Figure 8: Wildfire exposure by fire risk category given by First Street Foundation. A. Wildfire exposure risk estimates as given by First Street Foundation for each of the ten fire risk categories. **B.** Results of the wildfire exposure risk adjusted for exposure dependence of the next higher risk category. For the highest risk category, the exposure risk is the same.

We interpreted First Street Foundation's probabilities as the mean likelihood of exposure for a cluster of homes ($Exposure\ Risk_{FF}$). To model the interdependence of fire risk among neighboring groups of houses, we calculated ignition probabilities per fire risk category conditional on the wildfire exposure of the next higher category as the solution of the following equation:

$$(2)\quad Exposure\ Risk_{FF} = Wildfire\ Exposure_{FF\ High\ Category} * (Prob(Higher\ Category \mid Exposed)) + Wildfire\ Exposure_{FF} * (Prob(Higher\ Category \mid Not\ Exposed))$$

$$= Wildfire\ Exposure_{FF} * IncreaseFactor * (Prob(Higher\ Category \mid Exposed)) + Wildfire\ Exposure_{FF} * (Prob(Higher\ Category \mid Not\ Exposed))$$

We posited that the probability of wildfire exposure of any given risk category ($Wildfire\ Exposure_{FF}$) intensifies by a factor of three (*IncreaseFactor*) if an adjacent, higher risk category is exposed (*Prob(Higher Category | Exposed)*), compared to the case that the next higher category is not exposed (*Prob(Higher Category | Not Exposed)*).

The actual wildfire exposure is then calculated as the result of a Bernoulli trial, where the outcome is binary (i.e., exposed or not) for the entire risk category within a tract. Additionally, we modeled the influence of high roofing renewal rates on community fire risk to account for spill-over effects of hardened homes. We posited that a roofing renewal rate exceeding 50% within any risk category results in a reduction of wildfire exposure for the immediate lower risk category. This reduction was modeled to scale linearly from 0% at a 50% renewal rate to a maximum of 15% when the renewal rate reached 100%.

2. *Simulating the risk of destruction by a wildfire.* The likelihood of a house being destroyed if exposed to wildfire was simulated through a draw from a beta distribution with a mean value of 0.31 and sigma of 0.15, based on estimates from previous literature[39] that put the risk of destruction upon exposure to wildfire at approximately 31%. While their estimates do vary depending on building age, for the sake of simplicity, we did not make age-based distinctions in our model. We opted for a beta distribution over alternatives such as a normal distribution to more accurately capture the tail risk associated with major wildfire events. The actual risk of destruction was then calculated for both homes without and with new roofs per risk category, adjusting the latter's probability downward to account for the benefits of new roofs depending on the roof benefit scenario. We further accounted for spill-over effects of hardened homes by reducing the risk of destruction based on the roofing rate within a risk category when over 50% of homes in a category have renewed roofs. This reduction was modeled to scale linearly from 0% at a 50% renewal rate to a maximum of 10% when the renewal rate reached 100%.

3. *Calculating destroyed buildings and required roof renewal rate*. We calculated the average share of buildings which were expected to be destroyed by wildfire across different risk, roof benefit, and roof renewal rate scenarios. We then fitted a polynomial regression model to approximate the values between our chosen scenarios. The required roof renewal rate to reach a closure of the equity gap was then calculated as the intersection of the non-DAC destruction rate at their estimated roof renewal rate and the function given by the polynomial regression.

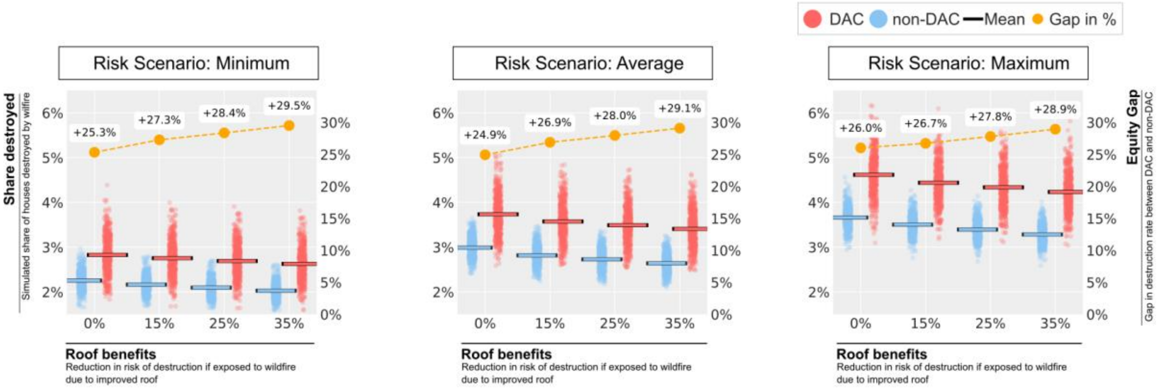

Supplementary Figure 9: Simulating the impact of the different roof renewal rates on the equity gap in terms of the share of destroyed buildings for DACs vs. non-DACs within the highest wildfire risk category (wildfire risk above the 90th percentile). Roofs are assumed to reduce the probability of being destroyed if being exposed to wildfire by 0 - 35%. Risk Scenarios vary between minimum risk (lower end of First Street Foundation exposure estimates) and maximum risk (higher end of exposure estimates).

Supplementary Note 12: Counterfactual Simulation - Buildings saved vs. Costs

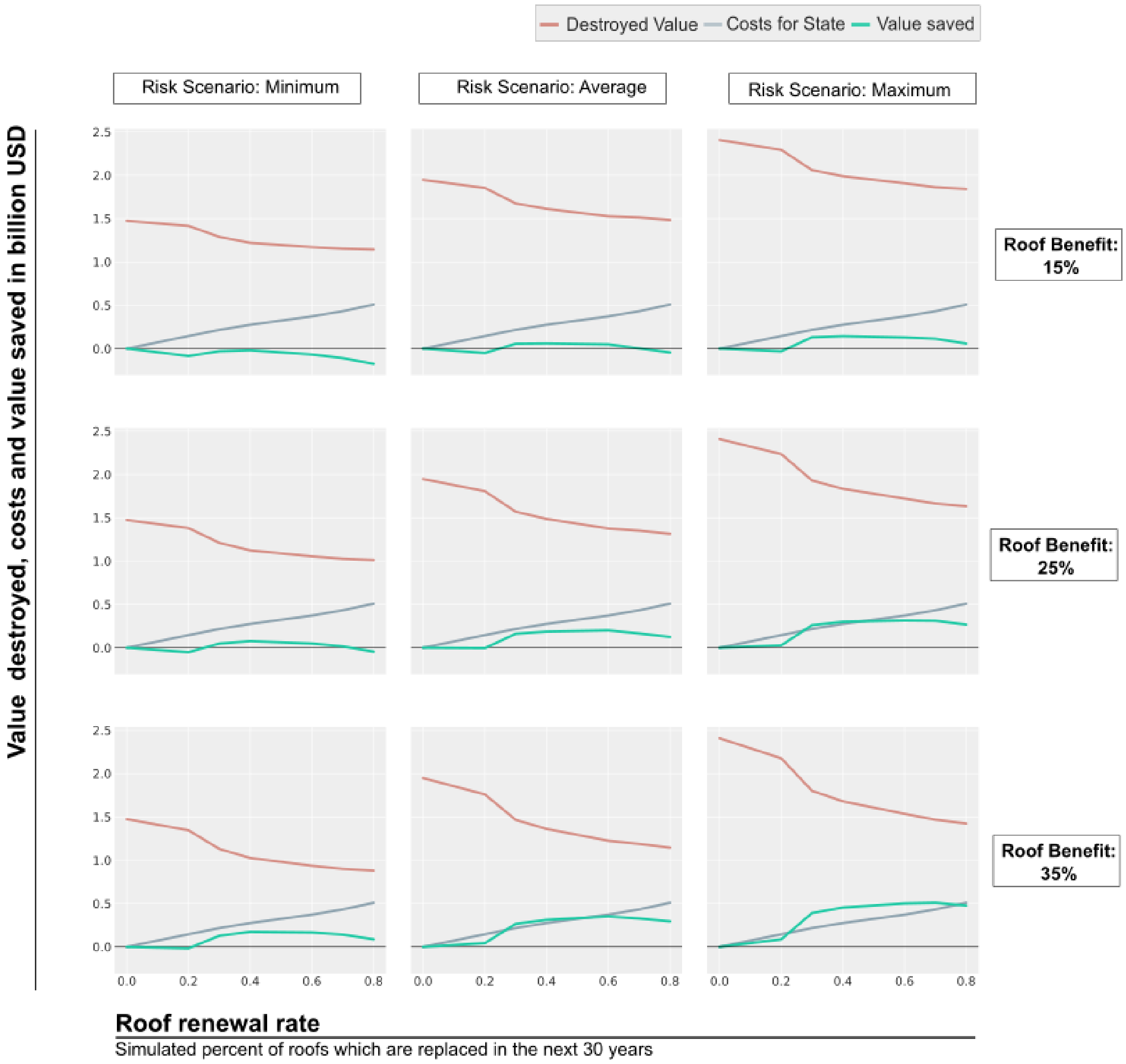

Supplementary Figure 10: Simulation of wildfire-induced damage in high-risk DAC tracts in California and value saved through roof renewals. Simulation of wildfire-induced damage within the highest risk DAC tracts in California in the next 30 years based on our counterfactual simulation (Supplementary Note 11). Value destroyed is calculated as the average destroyed number of houses multiplied by the average housing value in DAC tracts, as given by Zillow[56]. Costs for state are calculated as the number of roofs renewed at a given roof renewal rate, multiplied by the average roof renewal costs of 20,000 USD and an assumed 30% state coverage rate. Value saved is then the difference between the wildfire-induced value lost per roof renewal rate compared to the value lost in case of a roof renewal rate of 0.

Supplementary Note 13: Interaction of fire-resistant roof and construction year

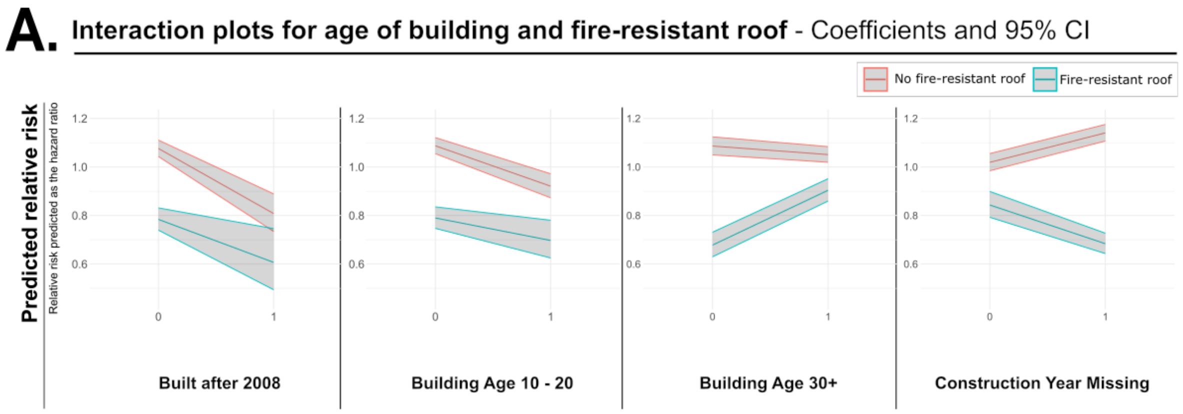

Supplementary Figure 11: Interaction plots for building age and fire resistant roof. Displayed values are the predicted hazard ratio based on a conditional logit regression, i.e. values below 1 signify a decrease in risk and values above 1 an increase in risk (Methods). Fire-resistance of roofs is set to 1 if all of these three criteria are fulfilled: Roof cover (is either asphalte, metal, concrete or tiles), vents (mesh screens > ⅛) and eaves (no eaves or enclosed eaves). n = 40,673 residential buildings exposed to wildfire. Statistical significance levels are * p<0.05, **p<0.01, and ***p<0.001.

Fire-resistant roofs reduce relative risk across all building age categories. To analyze the effects of fire-resistant roofing and its interaction with building age, we examined our dataset from the CAL FIRE DINS database, which includes 40,673 residential buildings, of which 33,689 were destroyed between 2013 and 2021 using a conditional logit regression (Methods), including all usual building and neighborhood related controls.

Fire-resistant roofs significantly reduce the predicted relative risk and likelihood of destruction across all age categories, with the lowest effect for buildings built after 2008, likely due to California regulations requiring newer buildings to use fire-resistant materials. Therefore, non-fire-resistant roofs are rarely observed in this category. The largest and most strongly significant interaction coefficient is observed when the building age is not observed in the database, possibly because buildings with unspecified ages may encompass a wider range of older structures with varied construction standards and materials, making them more susceptible to fire damage and thus benefiting more from fire-resistant roofs.

Supplementary Note 14: Using California CalEnviroScreen Score instead of Justice40 initiative measurement

An alternative measure to the disadvantaged community (DAC) indicator is the CalEnviroScreen Score, developed by the Office of Environmental Health Hazard Assessment (OEHHA) in California. This score identifies communities overburdened by pollution and socioeconomic factors such as low income or education levels. Unlike the binary DAC indicator, the CalEnviroScreen Score is a continuous variable, offering a more nuanced assessment. Additionally, the variables used in the CalEnviroScreen Score differ, with no direct consideration of fire and climate risk. Instead, it indirectly accounts for these risks by measuring current exposures, such as air quality, and includes factors like the population's sensitivity to diseases such as asthma[57].

To test the robustness of our results, we replaced the DAC indicator with the CalEnviroScreen Score to compare roof renewals per tract. While the average number of roof renewals per CalEnviroScreen Score (represented in quintiles for simplicity) shows a similar trend (fewer roof renewals in areas with higher scores), the effect is less pronounced than with the DAC indicator. However, when controlling for building and tract characteristics, the CalEnviroScreen Score remains negative and significant ($p<0.001$), demonstrating the robustness of our findings.

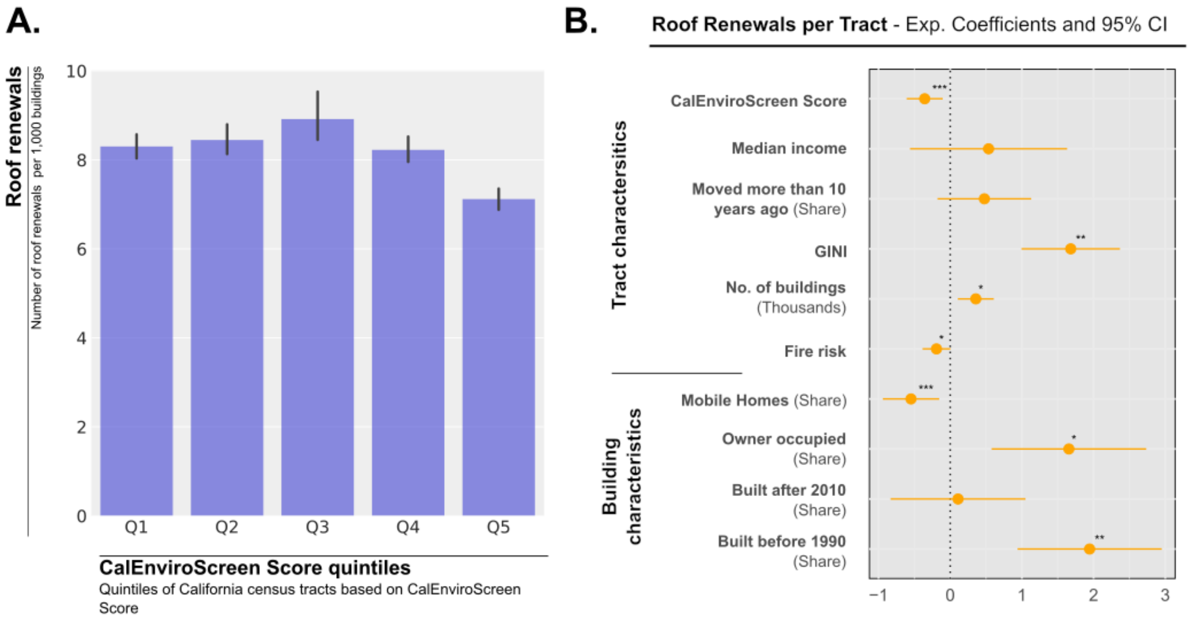

Supplementary Figure 12: Relationship of CalEnviroScreen Score and roof renewal rate in California census tracts. A. Average roof renewals per 1,000 households per CalEnviroScreen score quintiles in California. The sample includes n = 2,407 census tracts from California that were observed over a 9 year period. **B.** Exponentiated coefficients from a negative binomial count regression. The exponentiated coefficients displayed in this plot represent the percentage change in the number of roof renewals per census tract for a one-

unit change in the independent variables. In addition to the displayed independent variables, the model includes spatial and temporal fixed effects at the county and year level. The sample includes n = 2,407 census tracts from California that were observed over a 9 year period. All independent variables except for “No. of Buildings” are normalized to the [0,1] interval so the coefficients represent the difference in effect size when the corresponding variable is at its maximum (coded as 1) vs. minimum (coded as 0). Statistical significance levels are * $p<0.05$, ** $p<0.01$, and *** $p<0.001$.

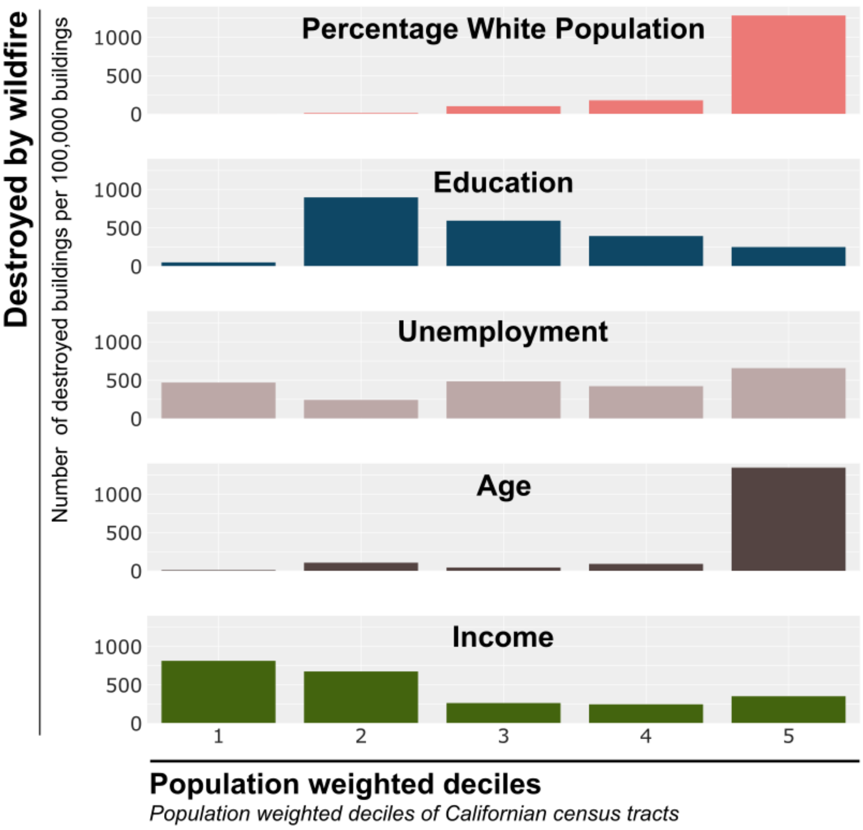

Supplementary Figure 13: Residential buildings destroyed by wildfire for different socioeconomic groups. Residential buildings destroyed by wildfire in California (2013-2021) by socio-demographic groups. Includes all residential buildings that are considered ‘destroyed’ based on CAL FIRE’s DINS database. Buildings are spatially merged with 2010 census tracts and population deciles are calculated using the population weighted median household income. The full sample includes n = *33,689* residential buildings. Measure of income deciles and destroyed buildings per 100,000 buildings includes 8,056 California census tracts (Methods).

Supplementary Note 15: Influence of large fire events

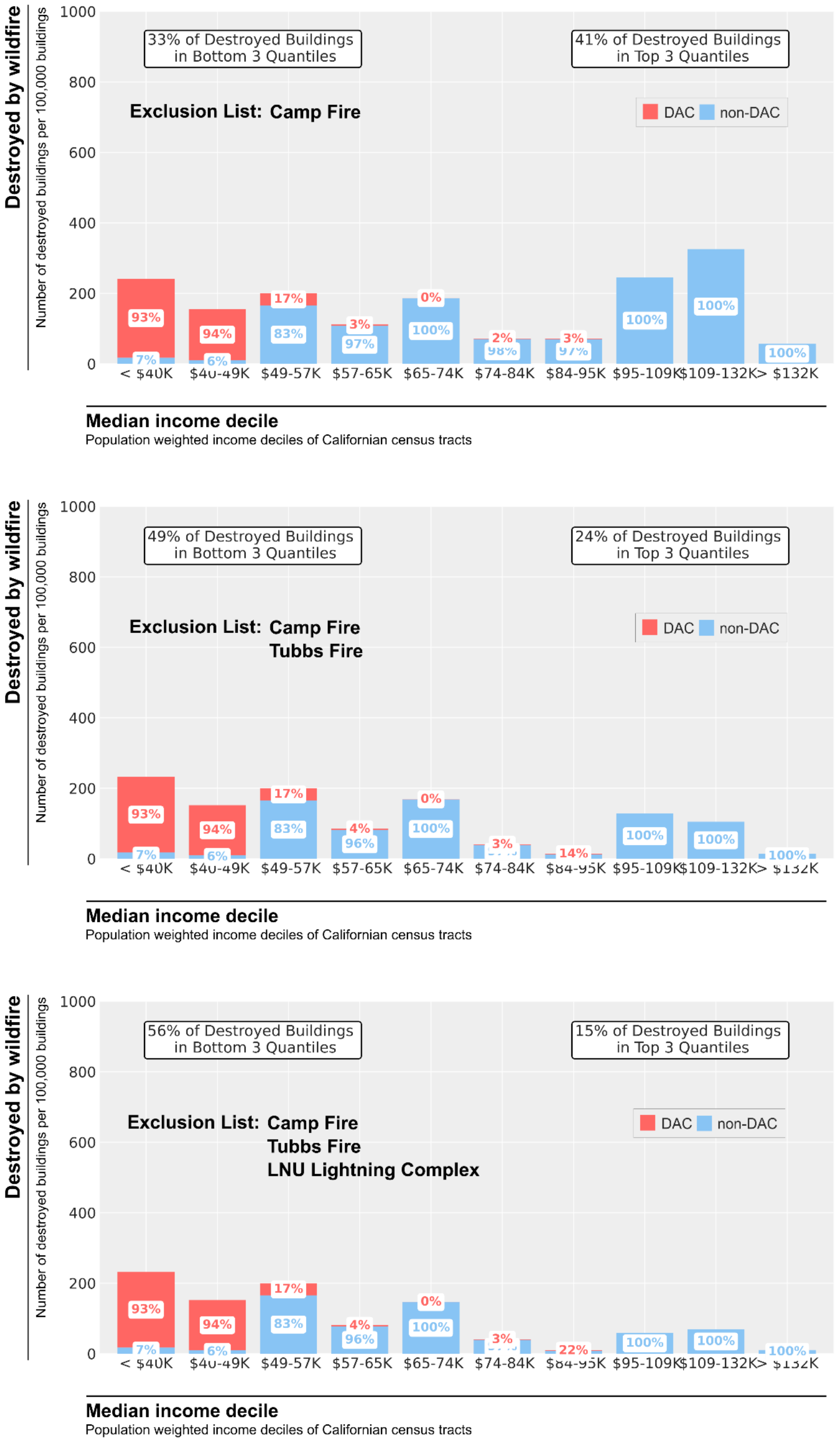

Supplementary Figure 14: Residential buildings destroyed by wildfire and influence of large fire events, gradual exclusion of largest fires. Residential buildings destroyed by wildfire in California (2013-2021) by median income groups. Includes all residential buildings that are considered 'destroyed' based on CAL FIRE's DINS database. Buildings are spatially merged with 2010 census tracts and income deciles are calculated using the population weighted median household income. Classification into disadvantaged communities (DAC) and non-disadvantaged communities (non-DAC) is based on the definition of the U.S. government's

Justice40 initiative[30], as implemented in its Climate and Economic Justice Screening Tool (CEJST). The full sample includes *n = 33,689* residential buildings. Measure of income deciles and destroyed buildings per 100,000 buildings includes 8,056 California census tracts (Methods). **A.** Exclusion of Camp Fire (n= 14,820) **B.** Exclusion of Camp Fire (n= 14,820) and Tubbs Fire (n=6,282) **C.** Exclusion of Camp Fire (n= 14,820), Tubbs Fire (n=6,282) and LNU Lightning Complex Fire (n=1,618)

We investigated the robustness of our results concerning the influence of individual wildfire events. Given that a few major fires account for a large portion of the observations in the CAL FIRE DINS Database, we excluded some of these events in our analyses to test robustness. Our findings indicate that the relationship between larger fire damage and lower-income communities remains consistent even when the three largest fires are excluded. However, this relationship is not evident when only the "Camp Fire" is excluded, likely because the "Camp Fire" disproportionately affected lower-income communities, whereas the "Tubbs Fire" had a greater impact on higher-income areas. Additionally, we re-analyzed the regression on the benefits of roof renewals and wildfire risk reduction. The differences were less pronounced compared to the descriptive analysis, and our main results remained intact (Supplementary Table 12).

Supplementary Note 16: Adjusting the roof renewal rates for the share of mobile homes

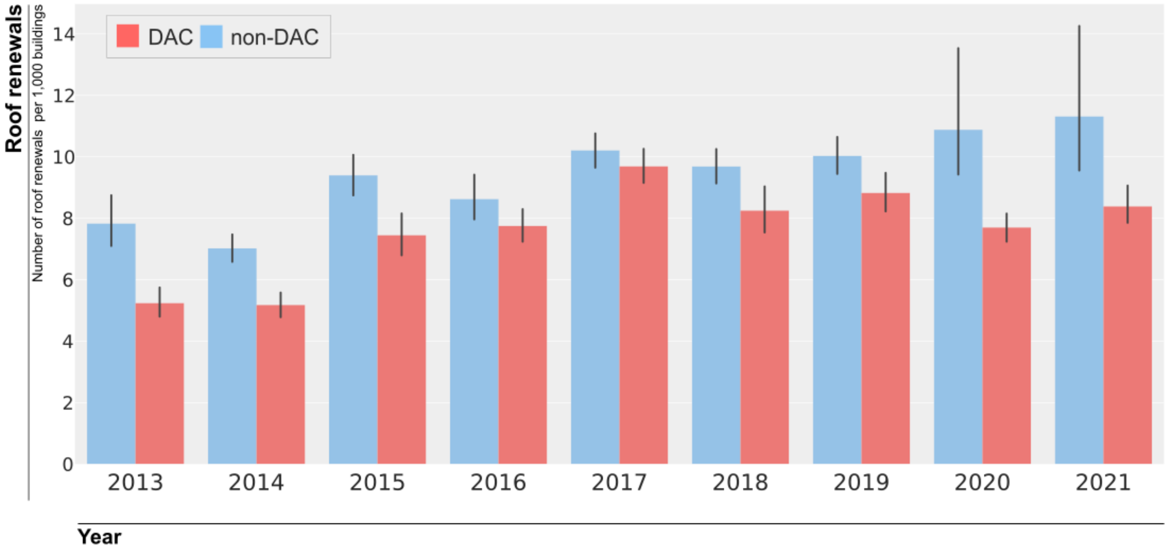

Supplementary Figure 15: Roof renewals per year in California within a census tract adjusted for mobile homes. The mean number of roof renewals per 1,000 buildings by year and disadvantaged community status (bars). 95% confidence intervals are indicated by black error bars. The number of houses per census tract is adjusted by the number of mobile homes to reflect differences in building stock. While the absolute numbers change, the overall pattern of less roof renewals in DAC tracts remains robust. The sample includes *n = 2,563* Californian census tracts.

Supplementary Note 17: Assessing the effect of the margin of error of the ACS median income estimates

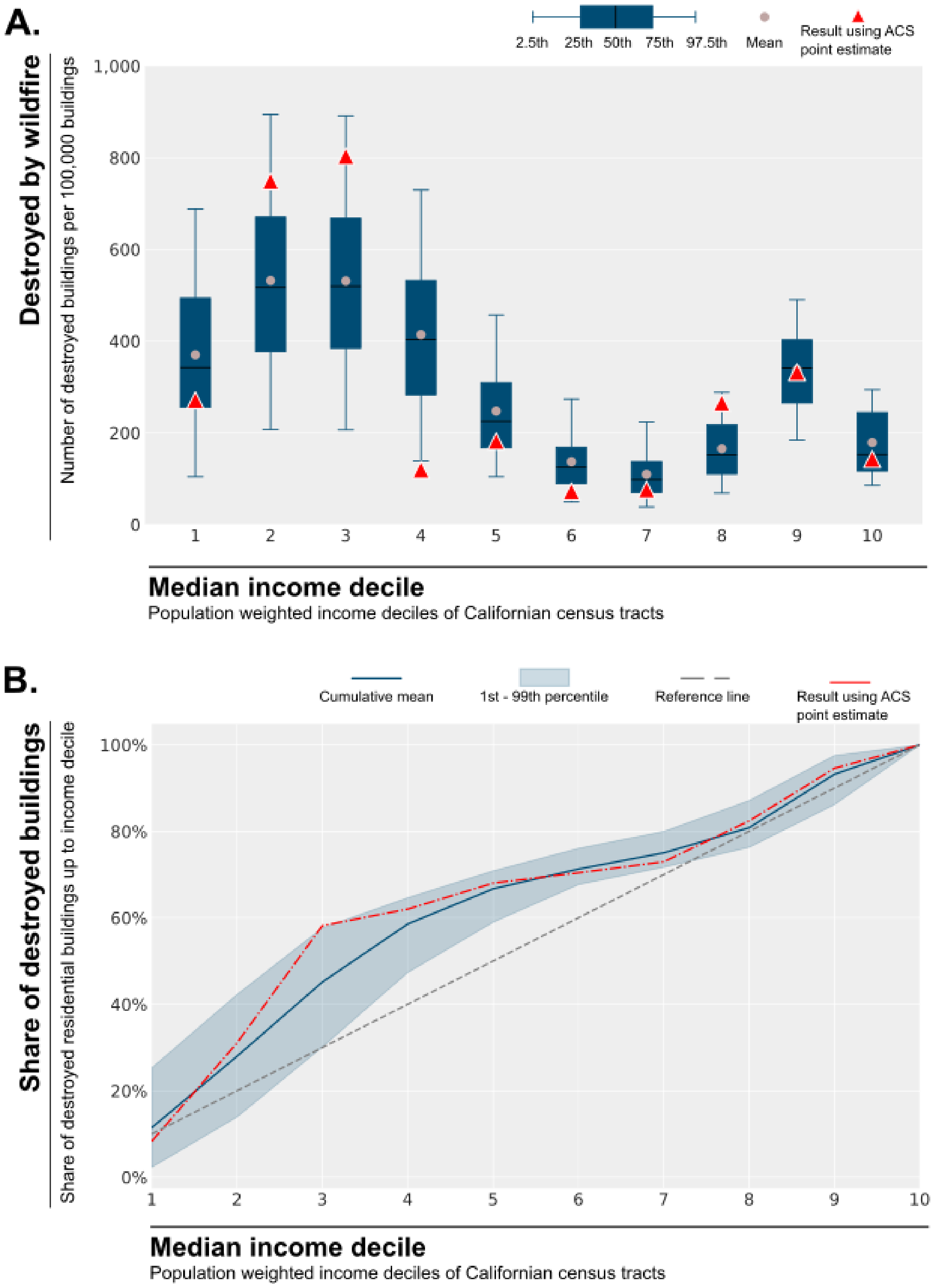

Supplementary Figure 16: Results of a probabilistic simulation with 10,000 runs A. Destruction as the number of destroyed residential buildings across income deciles, accounting for the margin of error in median household income as reported by the American Community Survey and categorizing Californian census tracts into population-weighted income deciles. The boxplots summarize the results of 10,000 simulation runs. Red triangles indicate the results using the ACS point estimates. The sample contains data

from 8,056 California census tracts and information on *n = 33,689* residential buildings that were destroyed by wildfire between 2013 and 2021. **B.** Cumulative distribution of share of destruction across income deciles. The distribution plot summarizes the results of 10,000 simulation runs. The red line indicates the results using the ACS point estimates. The sample contains data from 8,056 California census tracts and information on *n = 33,689* residential buildings that were destroyed by wildfire between 2013 and 2021.

To address the American Community Survey (ACS) estimates' margin of error (MoE) and verify the robustness of our findings, i.e. that the lowest three income deciles disproportionately suffered from wildfire-induced destruction of residential buildings, we implemented a probabilistic simulation of census tract median income. We simulated the median household income of a census tract by drawing from a tract-specific normal distribution, where the mean equals the ACS point estimate of median household income and the standard deviation equals the associated MoE divided by 1.645 (we divide by 1.645 because 90% of the probability mass of a normal distribution is contained within 1.645 standard deviations of the mean and the MoE represents a 90% confidence interval). For each simulation run, census tracts were then categorized into deciles based on their simulated median household income, and the destruction share for each income decile was calculated as the share of residential buildings destroyed by wildfire. We repeated this procedure 10,000 times to assess the sensitivity of our results to the MoE. In the lowest three income deciles, the average number of destroyed buildings per 100,000 residential units is more than double that of the other deciles (476 buildings compared to 227 in the middle four and 228 in the highest three) (Supplementary Fig. 16A). Given the margin of error (MoE) in ACS data, the confidence intervals in our simulation are quite large. This occurs because the MoE often matches the differences in median household income between deciles, causing tracts to easily shift between income groups. With only 274 tracts affected by wildfire damage, even a small deviation (one standard deviation) can move a tract into a different income decile. Moreover, there is a tendency toward the middle deciles in the simulations, as lower-income deciles can only move upwards.

A more robust approach to account for the between-decile shifts of tracts is to analyze the cumulative destruction share against a reference uniform distribution. The destruction share consistently exceeds the reference line, indicating a disproportionately higher share of buildings destroyed by wildfires in the lower-income deciles (Supplementary Fig. 16B). We therefore conclude that our main finding is robust to uncertainty introduced by the ACS' MoE.

Supplementary References

1. Rogers, R. W. A Protection Motivation Theory of Fear Appeals and Attitude Change1. *J. Psychol.* **91**, 93–114 (1975).
2. Milne, S., Sheeran, P. & Orbell, S. Prediction and Intervention in Health-Related Behavior: A Meta-Analytic Review of Protection Motivation Theory. *J. Appl. Soc. Psychol.* **30**, 106–143 (2006).
3. Tversky, A. & Kahnemann, D. JUDGMENT UNDER UNCERTAINTY: HEURISTICS AND BIASES. *Science* **185**, 1124–1131 (1974).
4. Bubeck, P., Botzen, W. J. W. & Aerts, J. C. J. H. A Review of Risk Perceptions and Other Factors that Influence Flood Mitigation Behavior: Review of Flood Risk Perceptions. *Risk Anal.* **32**, 1481–1495 (2012).
5. Larsen, L. N. D. *et al.* Risk perceptions and mitigation behaviors of residents following a near-miss wildfire. *Landsc. Urban Plan.* **207**, 104005 (2021).
6. Martin, I. M., Bender, H. & Raish, C. What Motivates Individuals to Protect Themselves from Risks: The Case of Wildland Fires. *Risk Anal.* **27**, 887–900 (2007).
7. Martin, W. E., Martin, I. M. & Kent, B. The role of risk perceptions in the risk mitigation process: The case of wildfire in high risk communities. *J. Environ. Manage.* **91**, 489–498 (2009).
8. Gordon, J. S., Luloff, A. & Stedman, R. C. A Multisite Qualitative Comparison of Community Wildfire Risk Perceptions. *J. For.* **110**, 74–78 (2012).
9. Gallagher, J. Learning About an Infrequent Event: Evidence from Flood Insurance Take-Up in the US. *Am. Econ. J. Appl. Econ.* 206–233 (2014) doi:10.2139/ssrn.3078097.
10. Brenkert-Smith, H., Champ, P. A. & Flores, N. Trying Not to Get Burned: Understanding Homeowners' Wildfire Risk–Mitigation Behaviors. *Environ. Manage.* **50**, 1139–1151 (2012).
11. Champ, P. A. & Brenkert-Smith, H. Is Seeing Believing? Perceptions of Wildfire Risk Over Time: Is Seeing Believing? Perceptions of Wildfire Risk Over Time. *Risk Anal.* **36**, 816–830 (2016).
12. McCaffrey, S. Crucial factors influencing public acceptance of fuels treatments. (2009).
13. McCaffrey, S., Stidham, M., Toman, E. & Shindler, B. Outreach Programs, Peer Pressure, and Common Sense: What Motivates Homeowners to Mitigate Wildfire Risk? *Environ. Manage.* **48**, 475–88 (2011).
14. Wolters, E. A., Steel, B. S., Weston, D. & Brunson, M. Determinants of residential Firewise behaviors in Central Oregon. *Soc. Sci. J.* **54**, 168–178 (2017).
15. Brown, T. C. & Kroll, S. Inequality hinders group efforts to avoid environmental disasters. *Q Open* **1**, qoab006 (2021).
16. Kyle, G. T., Theodori, G. L., Absher, J. D. & Jun, J. The Influence of Home and Community Attachment on Firewise Behavior. *Soc. Nat. Resour.* **23**, 1075–1092 (2010).

17. Ojerio, R., Moseley, C., Lynn, K. & Bania, N. Limited Involvement of Socially Vulnerable Populations in Federal Programs to Mitigate Wildfire Risk in Arizona. *Nat. Hazards Rev.* **12**, 28–36 (2011).
18. Thomas, D., Butry, D., Gilbert, S., Webb, D. & Fung, J. *The Costs and Losses of Wildfires: A Literature Survey*. NIST SP 1215 (2017) doi:10.6028/NIST.SP.1215;
19. Anderson, S., Plantinga, A. & Wibbenmeyer, M. Inequality in Agency Responsiveness: Evidence from Salient Wildfire Events. *J. Polit.* **85**, 625–639 (2023).
20. Sánchez, J. J., Holmes, T. P., Loomis, J. & González-Cabán, A. Homeowners willingness to pay to reduce wildfire risk in wildland urban interface areas: Implications for targeting financial incentives. *Int. J. Disaster Risk Reduct.* **68**, 102696 (2022).
21. Bakkensen, L. & Barrage, L. *Flood Risk Belief Heterogeneity and Coastal Home Price Dynamics: Going Under Water?* w23854 http://www.nber.org/papers/w23854.pdf (2017) doi:10.3386/w23854.
22. McCoy, S. & Walsh, R. Wildfire risk, salience & housing demand. *J. Environ. Econ. Manag.* **91**, 203--228 (2018).
23. Dupey, L. N. & Smith, J. An Integrative Review of Empirical Research on Perceptions and Behaviors Related to Prescribed Burning and Wildfire in the United States. *Environ. Manage.* **61**, (2018).
24. Radeloff, V. C. *et al.* Rapid growth of the US wildland-urban interface raises wildfire risk. *Proc. Natl. Acad. Sci.* **115**, 3314–3319 (2018).
25. Modaresi Rad, A. *et al.* Human and infrastructure exposure to large wildfires in the United States. *Nat. Sustain.* **6**, 1343–1351 (2023).
26. Davies, I. P., Haugo, R. D., Robertson, J. C. & Levin, P. S. The unequal vulnerability of communities of color to wildfire. *PLOS ONE* **13**, e0205825 (2018).
27. Gabbe, C. J., Pierce, G. & Oxlaj, E. Subsidized Households and Wildfire Hazards in California. *Environ. Manage.* **66**, 873–883 (2020).
28. Wigtil, G. *et al.* Places where wildfire potential and social vulnerability coincide in the coterminous United States. *Int. J. Wildland Fire* **25**, 896 (2016).
29. Coughlan, M. R., Ellison, A. & Cavanaugh, A. Social Vulnerability and Wildfire in the Wildland-Urban Interface. *Northwest Fire Sci. Consort.* (2019).
30. The White House. Justice40 Initiative | Environmental Justice. *The White House* https://www.whitehouse.gov/environmentaljustice/justice40/ (2022).
31. Masri, S., Scaduto, E., Jin, Y. & Wu, J. Disproportionate Impacts of Wildfires among Elderly and Low-Income Communities in California from 2000–2020. *Int. J. Environ. Res. Public. Health* **18**, 3921 (2021).
32. Palaiologou, P., Ager, A. A., Nielsen-Pincus, M., Evers, C. R. & Day, M. A. Social vulnerability to large wildfires in the western USA. *Landsc. Urban Plan.* **189**, 99–116 (2019).

33. Hamann, M. *et al.* Inequality and the Biosphere. *Annu. Rev. Environ. Resour.* **43**, 61–83 (2018).
34. de Diego, J., Rúa, A. & Fernández, M. Vulnerability Variables and Their Effect on Wildfires in Galicia (Spain). A Panel Data Analysis. *Land* **10**, 1004 (2021).
35. Burke, M. *et al.* Exposures and behavioural responses to wildfire smoke. *Nat. Hum. Behav.* **6**, 1351–1361 (2022).
36. Wen, J. & Burke, M. Lower test scores from wildfire smoke exposure. *Nat. Sustain.* **5**, 947–955 (2022).
37. Hazlett, C. & Mildenberger, M. Wildfire Exposure Increases Pro-Environment Voting within Democratic but Not Republican Areas. *Am. Polit. Sci. Rev.* **114**, 1359–1365 (2020).
38. Lim, T., Kim, T. T. & Kim, S. Y. Consumer Response to Climate Change: Wildfire Smoke and Sustainable Product Choice. *SSRN Electron. J.* (2023) doi:10.2139/ssrn.4439697.
39. Baylis, P. & Boomhower, J. Mandated vs. voluntary adaptation to natural disasters: The case of U.S. wildfires. Preprint at (2022).
40. Baylis, P. & Boomhower, J. *Moral Hazard, Wildfires, and the Economic Incidence of Natural Disasters*. w26550 http://www.nber.org/papers/w26550.pdf (2019) doi:10.3386/w26550.
41. Blanchi, R., Leonard, J. & Leicester, R. Lessons learnt from post-fire surveys at the urban interface in Australia. *For. Ecol. Manag. - For. ECOL MANAGE* **234**, (2006).
42. Hakes, R. S. P., Caton, S. E., Gorham, D. J. & Gollner, M. J. A Review of Pathways for Building Fire Spread in the Wildland Urban Interface Part II: Response of Components and Systems and Mitigation Strategies in the United States. *Fire Technol.* **53**, 475–515 (2017).
43. ReadyforWildfire. Wildfire Action Plan. https://www.fire.ca.gov/what-we-do/-/media/calfire-website/about/communications/cal-fire-ready-set-go-brochure-final-files-v4-print.pdf (n.d.).
44. CAL FIRE. Home Hardening. *https://www.fire.ca.gov/home-hardening* https://view.genial.ly/63f4efb6c5a3ab0010a62297.
45. Papathoma-Köhle, M. *et al.* A wildfire vulnerability index for buildings. *Sci. Rep.* **12**, 6378 (2022).
46. Syphard, A. D., Brennan, T. J. & Keeley, J. E. The importance of building construction materials relative to other factors affecting structure survival during wildfire. *Int. J. Disaster Risk Reduct.* **21**, 140–147 (2017).
47. Pagni, P. & Woycheese, J. P. Fire spread by brand spotting. *Proc. Fifteenth Meet. UJNR Panel Fire Res. Saf.* **2**, 373–380 (2000).
48. Barret, K. Wood roofs are a $6 billion wildfire problem. *Headwaters Economics* https://headwaterseconomics.org/natural-hazards/wood-roofs-wildfire/ (2022).
49. CAL FIRE. Statistics | Top 20 Most Destructive California Wildfires. *Statistics* https://www.fire.ca.gov/our-impact/statistics (2023).
50. Barret, K. Wildfires destroy thousands of structures each year. *Headwaters Economics* https://headwaterseconomics.org/natural-hazards/structures-destroyed-by-wildfire/ (2020).

51. US Census Bureau. TIGER/Line Geodatabases. *Census.gov* https://www.census.gov/geographies/mapping-files/time-series/geo/tiger-geodatabase-file.html.

52. Childs, M. L. *et al.* Daily Local-Level Estimates of Ambient Wildfire Smoke PM2.5 for the Contiguous US. *Environ. Sci. Technol.* **56**, 13607–13621 (2022).

53. Miller, S., Johnson, N. & Wherry, L. R. Medicaid And Mortality: New Evidence From Linked Survey And Administrative Data. *Q. J. Econ.* **136**, 1783–1829 (2021).

54. First Street Foundation. Climate Risk Publicly Available Data. *First Street Foundation* https://firststreet.org/documentation.

55. California Department of Finance: Department of Finance | State of California. *Demographics* https://dof.ca.gov/forecasting/demographics/projections/.

56. Housing Data. *Zillow* https://www.zillow.com/research/data/.

57. Admin, O. Indicators Overview. *OEHHA* https://oehha.ca.gov/calenviroscreen/indicators (2015).
