# Supplementary material for: Roof renewal disparities widen the equity gap in residential wildfire protection
Source: Nat Commun. 2025 Jan 7;16:463. doi: 10.1038/s41467-024-55705-w (PMC11707273; doi:10.1038/s41467-024-55705-w)
Supplement: Supplementary file 2 — Reporting Summary [file 41467_2024_55705_MOESM2_ESM.pdf]

nature portfolio

Corresponding author(s): Sebastian Reining

Last updated by author(s): Oct 17, 2024

Reporting Summary

Nature Portfolio wishes to improve the reproducibility of the work that we publish. This form provides structure for consistency and transparency in reporting. For further information on Nature Portfolio policies, see our Editorial Policies and the Editorial Policy Checklist.

Statistics

For all statistical analyses, confirm that the following items are present in the figure legend, table legend, main text, or Methods section.

n/a	Confirmed	
☐	☒	The exact sample size (*n*) for each experimental group/condition, given as a discrete number and unit of measurement
☐	☒	A statement on whether measurements were taken from distinct samples or whether the same sample was measured repeatedly
☐	☒	The statistical test(s) used AND whether they are one- or two-sided<br>*Only common tests should be described solely by name; describe more complex techniques in the Methods section.*
☐	☒	A description of all covariates tested
☐	☒	A description of any assumptions or corrections, such as tests of normality and adjustment for multiple comparisons
☐	☒	A full description of the statistical parameters including central tendency (e.g. means) or other basic estimates (e.g. regression coefficient) AND variation (e.g. standard deviation) or associated estimates of uncertainty (e.g. confidence intervals)
☐	☒	For null hypothesis testing, the test statistic (e.g. *F*, *t*, *r*) with confidence intervals, effect sizes, degrees of freedom and *P* value noted<br>*Give P values as exact values whenever suitable.*
☒	☐	For Bayesian analysis, information on the choice of priors and Markov chain Monte Carlo settings
☒	☐	For hierarchical and complex designs, identification of the appropriate level for tests and full reporting of outcomes
☒	☐	Estimates of effect sizes (e.g. Cohen's *d*, Pearson's *r*), indicating how they were calculated

Our web collection on statistics for biologists contains articles on many of the points above.

Software and code

Policy information about availability of computer code

Data collection	Data, especially building permit data were obtained through Freedom of Information Act Requests. No softeware was used to to collect data. Building Permits were then filtered, geo-located and identified as "roofing permit" using Python Jupyter notebooks
Data analysis	Python Jupyter Notebooks were used to perform data cleaning, data preparation, generation of figures and descriptive statistics.<br>R version 4.2.3 (2023-03-15) was used to perform the regression analysis and figure generation. All Code is available through the Open Science Framework at https://osf.io/f8g94/?view_only=3f12a54e48a2442c876810fd1ad73156

For manuscripts utilizing custom algorithms or software that are central to the research but not yet described in published literature, software must be made available to editors and reviewers. We strongly encourage code deposition in a community repository (e.g. GitHub). See the Nature Portfolio guidelines for submitting code & software for further information.

Data

Policy information about availability of data

All manuscripts must include a data availability statement. This statement should provide the following information, where applicable:

- Accession codes, unique identifiers, or web links for publicly available datasets
- A description of any restrictions on data availability
- For clinical datasets or third party data, please ensure that the statement adheres to our policy

All of the data applied in our study were either acquired from public sources or were obtained via Freedom of Information Act and public records requests. CAL

FIRE's Damage Inspection Database (DINS) is available at (https://www.fire.ca.gov/about/resources/ california-public-records) (Request number R006742-013123). American Community Survey (ACS) 5-year estimates are available at the Census Bureau (https://data.census.gov/.). The Climate and Environmental Justice dataset is available at the U.S. Council on Environmental Quality (https://www.whitehouse.gov/ environmentaljustice/justice40/). Building permits and assessor parcel maps were obtained from local authorities through open data portals or public requests. CAL FIRE's repository for fire perimeters is available through their GIS datacenter (https://frap.fire.ca.gov/mapping/gis-data/). Wildfire risk estimates from First Street are available for public (https://firststreet.org/data-access/public-access/). Firewise communities data is available through the NFPA. (https://www.nfpa.org/Public-Education/ Fire-causes-and-risks/Wildfire/Firewise-USA).

The data for replicating the analyses presented in this study is deposited in the Open Science Framework under accession code https://osf.io/f8g94/?view_only=3f12a54e48a2442c876810fd1ad73156

Research involving human participants, their data, or biological material

Policy information about studies with human participants or human data. See also policy information about sex, gender (identity/presentation), and sexual orientation and race, ethnicity and racism.

Reporting on sex and gender	All data is aggregated at the census tract level. Information on sex/gender are not included
Reporting on race, ethnicity, or other socially relevant groupings	All data is aggregated at the census tract level. We do not calculate heterogeneous effect of socially relevant groupings apart from the indicators<br>income and "disadvantaged communities", which includes age / education but not race or ethinicity (apart from tribal areas).
Population characteristics	All variables are aggregated at the census tract level. Tract-level covariates, including education, income, economic and social disadvantaged<br>indicators were used to estimate heterogeneous effects.
Recruitment	Building permits were either obtained through Freedom of Information Act Requests to local authorities or can be directly downloaded from<br>City / County Open Data Websites.
Ethics oversight	Only aggreggated data was used for analysis. Approval of study protocol was not required

Note that full information on the approval of the study protocol must also be provided in the manuscript.

Field-specific reporting

Please select the one below that is the best fit for your research. If you are not sure, read the appropriate sections before making your selection.

☐ Life sciences ☒ Behavioural & social sciences ☐ Ecological, evolutionary & environmental sciences

For a reference copy of the document with all sections, see nature.com/documents/nr-reporting-summary-flat.pdf

Behavioural & social sciences study design

All studies must disclose on these points even when the disclosure is negative.

Study description	Quantitative quasi-experimental method using observational data
Research sample	The data used in this analysis consists of building permits for California households
Sampling strategy	We used all Californian counties for which our Freedom of Information Act requests resulted in a (useable) database.
Data collection	Our study utilized data from public sources and records obtained via Freedom of Information Act requests, encompassing seven key datasets:<br>(1) CAL FIRE's Damage Inspection Database (DINS) for details on structures affected by California wildfires (2013-2022); (2) Socioeconomic data from the American Community Survey (ACS) 5-year estimates;<br>(3) Climate and Environmental Justice dataset by the U.S. Council on Environmental Quality for classifying census tracts; (4) Building permits and assessor parcel maps for tracking roof renewals across 2,200 California census tracts (2013-2021);<br>(5) CAL FIRE's repository for fire perimeters; (6) Wildfire risk estimates from First Street Foundation for future exposure simulations; and (7) NFPA data for the establishment and location of Firewise communities.
Timing	Building Permits were collected from 2013 - 2021, fire perimeters were used from 2011 - 2021 and the destroyed buildings wered analyzed in a timeframe from 2013 - 2021
Data exclusions	We excluded all building permits prior to 2013 to ensure a balanced panel across counties<br>- Depending on county, between 10-30% of overall building permits were dropped due to incomplete data. Especially if no geoloaction is possible<br>- 304 tracts were excluded where less than 5 roofing permits in the course of 8 years where observed to minimize observations of partial tracts as tracts do not always correspond to city boundaries
Non-participation	Experiments were not conducted for this study. Code and data are available to ensure reproducibilty

Randomization	The authors did not conduct randomization of the analysis. Instead, a quasi-experimental study setting was used to analyze observational data. For the simulation of wildfire exposure, a seed was set in all python scrits to ensure reproducibilty in case a random variable is used

Reporting for specific materials, systems and methods

We require information from authors about some types of materials, experimental systems and methods used in many studies. Here, indicate whether each material, system or method listed is relevant to your study. If you are not sure if a list item applies to your research, read the appropriate section before selecting a response.

Materials & experimental systems

n/a	Involved in the study	
☒	☐	Antibodies
☒	☐	Eukaryotic cell lines
☒	☐	Palaeontology and archaeology
☒	☐	Animals and other organisms
☒	☐	Clinical data
☒	☐	Dual use research of concern
☒	☐	Plants

Methods

n/a	Involved in the study	
☒	☐	ChIP-seq
☒	☐	Flow cytometry
☒	☐	MRI-based neuroimaging

Plants

Seed stocks	No plants were used in this study.
Novel plant genotypes	No plants were used in this study.
Authentication	No plants were used in this study.
